# Cellular crosstalk regulates the aqueous humor outflow pathway and provides new targets for glaucoma therapies

**DOI:** 10.1038/s41467-021-26346-0

**Published:** 2021-10-18

**Authors:** Benjamin R. Thomson, Pan Liu, Tuncer Onay, Jing Du, Stuart W. Tompson, Sol Misener, Raj R. Purohit, Terri L. Young, Jing Jin, Susan E. Quaggin

**Affiliations:** 1 Division of Nephrology and Hypertension, Northwestern University Feinberg School of Medicine, Chicago, IL USA; 2 The Feinberg Cardiovascular and Renal Research Institute, Chicago, IL USA; 3 Department of Ophthalmology and Visual Sciences, University of Wisconsin–Madison, Madison, WI USA

**Keywords:** Angiogenesis, Recombinant protein therapy, Glaucoma

## Abstract

Primary congenital glaucoma (PCG) is a severe disease characterized by developmental defects in the trabecular meshwork (TM) and Schlemm’s canal (SC), comprising the conventional aqueous humor outflow pathway of the eye. Recently, heterozygous loss of function variants in *TEK* and *ANGPT1* or compound variants in *TEK*/*SVEP1* were identified in children with PCG. Moreover, common variants in *ANGPT1*and *SVEP1* have been identified as risk alleles for primary open angle glaucoma (POAG) in GWAS studies. Here, we show tissue-specific deletion of *Angpt1* or *Svep1* from the TM causes PCG in mice with severe defects in the adjacent SC. Single-cell transcriptomic analysis of normal and glaucomatous *Angpt1* deficient eyes allowed us to identify distinct TM and SC cell populations and discover additional TM-SC signaling pathways. Furthermore, confirming the importance of angiopoietin signaling in SC, delivery of a recombinant ANGPT1-mimetic promotes developmental SC expansion in healthy and *Angpt1* deficient eyes, blunts intraocular pressure (IOP) elevation and RGC loss in a mouse model of PCG and lowers IOP in healthy adult mice. Our data highlight the central role of ANGPT1-TEK signaling and TM-SC crosstalk in IOP homeostasis and provide new candidates for SC-targeted glaucoma therapy.

## Abstract (web summary)

Primary congenital glaucoma (PCG) is characterised by increased intraocular pressure, and variants in *ANGPT1*, or *SVEP1* have been identified as risk alleles. Here, the authors show that deletion of these genes induces glaucoma in mice, and that activation of ANGPT1-TEK signaling ameliorates disease progression in mouse models.

## Introduction

Glaucoma is a progressive neurodegenerative disease with no cure, affecting over 60 million individuals worldwide and leaving 8 million blind^1^. Current therapy is supportive and focused on the reduction of intraocular pressure (IOP), a primary risk factor for glaucoma progression. IOP homeostasis is regulated by the rates of aqueous humor secretion from the ciliary body and drainage through outflow pathways in the iridocorneal angle. The majority of outflow is conducted through the conventional route, comprised of the trabecular meshwork (TM) and Schlemm’s canal (SC), a large lymphatic/vascular hybrid vessel adjacent to the iridocorneal angle^2–5^. Patients with high-pressure glaucoma, including primary congenital glaucoma (PCG), a severe, early onset form of the disease, exhibit defects in the conventional pathway, which lead to decreased aqueous humor outflow and elevated IOP^6–8^. Current drugs, including prostaglandin analogs, carbonic anhydrase inhibitors, β-adrenergic antagonists, α_2_-adrenergic agonists, and Rho-kinase inhibitors are generally effective at lowering IOP and slowing disease progression. However, even with treatment many patients do not reach IOP targets and more than 30% require combination therapy with two or more IOP-lowering drugs^9,10^. Moreover, as not all available drugs are suitable for every patient, there is an unmet need for new therapeutic options with complementary mechanisms to be used alone or to supplement existing therapies.

The angiopoietin (ANGPT)-TEK (tunica interna endothelial cell kinase, also known as TIE2) system is an endothelial growth factor pathway comprised of the receptor tyrosine kinase TEK, which is highly expressed by SC endothelial cells^4,11,12^, and its ligands, the angiopoietins. Heterozygous loss of function variants in *TEK* or its primary ligand *ANGPT1* have been linked to PCG in children^13–15^, and *ANGPT1* and *ANGPT2* have been associated with primary open-angle glaucoma (POAG) in adults^16,17^. ANGPT1-TEK signaling is required for SC development in mice^13,14,18^, suggesting a potential mechanism for IOP elevation. In addition, the pathway is an essential regulator of IOP homeostasis in adult mice and nonhuman primates, which rapidly develop ocular hypertension and glaucoma after inhibition of ANGPT-TEK signaling^18,19^.

In parallel with ANGPT-TEK signaling, SC and the TM are regulated by numerous pathways, many of which are likely to regulate outflow and provide IOP-lowering therapeutic targets. However, while numerous glaucoma or IOP-associated genes have been identified by genome-wide association studies (GWAS) and other large-scale genetics approaches, the expression pattern and functions of the products encoded by these genes are often unknown, making it difficult to prioritize candidates for study and focused therapeutic exploitation. One such gene is *SVEP1* (Sushi, von Willebrand factor type A, EGF, and pentraxin domain-containing protein 1), encoding a large extracellular matrix protein also known as Polydom which is expressed in the TM and has been reported to bind ANGPT1 in vitro^20,21^. SVEP1 is essential for lymphatic development and was linked to PCG via a large family containing five generations of affected individuals also harboring a heterozygous loss of function variant in *TEK*^22^ and has been associated with POAG in a large multi-ethnic cohort^23^.

Using mouse models, we show that deletion of *Angpt1* or *Svep1* from the neural crest tissues giving rise to the TM leads to major defects in the adjacent SC, ocular hypertension and phenotypes similar to PCG. These studies demonstrate the importance of TM-SC crosstalk and confirm that TM-expressed molecules are essential drivers of SC development and function. We then exploit the neural crest-specific *Angpt1* knockout model by performing single-cell transcriptomic analysis on wild-type and glaucomatous *Angpt1*-deficient eyes. This approach allowed us to identify TM and SC-specific molecules and networks that are potentially responsible for TM-SC reciprocal signaling in development and disease. The expression of known endothelial signaling molecules in the TM reinforces the notion that TM-SC crosstalk is an essential regulator of IOP homeostasis. This dataset provides a valuable resource for future hypothesis generation and therapeutic target selection. Finally, as proof of concept, we show that delivery of one such TM-expressed molecule, an ANGPT1 mimetic, enhances developmental SC formation, blunts IOP elevation and RGC loss in *Angpt1* deficient eyes and lowers IOP in healthy adult mice.

## Results

### Angpt1 is expressed by neural crest-derived cells in the anterior chamber

**Fig. 1:**
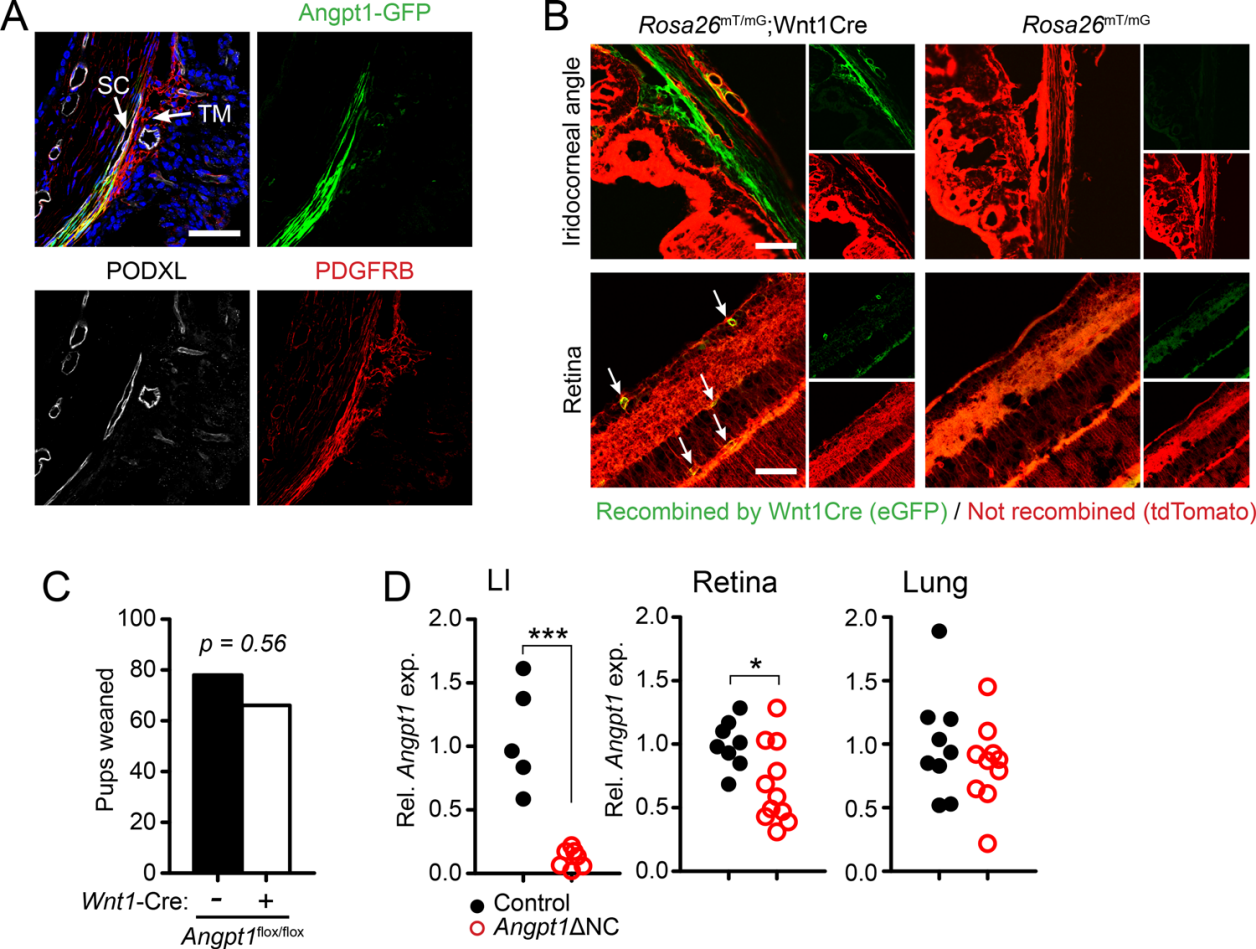
Ocular *Angpt1*-expressing tissues are derived from the neural crest. **A** *Angpt1*-GFP staining showing expression in PDGFRB-positive tissues of the uveal tract. As expected, no *Angpt1* expression was detected in PODXL-positive endothelial cells. Panel shown is a representative section from a group of four littermate animals. **B** Visualized using the *Rosa26*^mT/mG^ reporter mouse line, cre-mediated recombination in the neural crest lineage using *Wnt1*-Cre recapitulated *Angpt1* expression in the trabecular meshwork and uveal tissues. In the retina, recombination was limited to pericytes (white arrows). **C** Neural crest specific knockout (*Angpt1*ΔNC) mice are born normally in the predicted Mendalian ratio. *p* = 0.56 as determined by two-tailed Fisher’s exact test. Pups weaned: Control, *n* = 78, *Angpt1*ΔNC, *n* = 66. **D** As measured in 12-week-old mice using quantitative rtPCR, *Angpt1* mRNA expression was dramatically reduced in the limbus/iridocorneal angle region (LI) of *Angpt1*ΔNC mice (*p* = 0.0001). A moderate reduction in expression was observed in the retina (*p* = 0.0203), consistent with retinal *Angpt1* expression by both NC and non-NC-derived cells. In contrast, *Angpt1* expression in solid organs such as the lung was not altered (*p* = 0.368). Each point represents expression measured in an individual animal (two pooled eyes). Control, *n* = 5, *Angpt1*ΔNC, *n* = 7 (limbus) and control, *n* = 8, *Angpt1*ΔNC *n* = 11 (retina and lung). Scale bars represent 50 μm (A,B). *p < 0.05, ***p < 0.001 as determined by two-tailed Student’s *t*-test. Df_LI_ = 10, Df_retina, lung_ = 17.

Using transgenic reporter mice, we and others have described high levels of *Angpt1* expression in the uveal tissues of the eye, including the choroid and TM (Fig. 1A)^24,25^. Outside of the neural retina, ocular *Angpt1*-GFP expression was confined to tissues of neural crest origin, which include the TM and other cells of the uvea but not ocular endothelium, RPE, or ciliary epithelium. However, expression of native ANGPT1 remains poorly explored and previous studies with genetic knockouts have focused on whole-body inducible models. To confirm the neural crest origin of *Angpt1*-expressing tissues in mouse eyes, we crossed a previously validated *Wnt1*-Cre mouse line (B6.Cg-H2az2^Tg(Wnt1-cre)11Rth  Tg^(Wnt1-GAL4)11Rth/J)^26^ with *Rosa26*^mTmG^ (Gt(ROSA)26Sor^tm4(ACTB-tdTomato,-EGFP)Luo^/J) reporter mice which ubiquitously express tdTomato and activate eGFP only after Cre-mediated recombination^27^. As expected, in *Wnt1*-cre expressing mice we found robust recombination in tissues reported to be neural crest derived, including the TM, sclera, and retinal pericytes but not in mesoderm-derived endothelium or neuroepithelium-derived structures such as the retinal pigment epithelium (RPE), neural retina or ciliary body (Fig. 1B)^28–30^. A strong similarity was observed between the patterning of *Angpt1*-GFP expressing and *Wnt1*-Cre-recombined cells, especially in the TM and uvea.

To validate these findings in detail, we next generated neural crest-specific *Angpt1* knockout mice (hereafter *Angpt1*ΔNC) by crossing *Wnt1*-Cre expressing mice with a previously described *Angpt1*-floxed line^31^. *Angpt1*ΔNC mice were born normally and predicted Mendelian ratios were observed when genotyped at 3 weeks of age (Fig. 1C, *p* = 0.56 compared to a null hypothesis of equal genotype proportions using Fisher’s exact test). To confirm Cre-mediated excision, eyes were obtained from a cohort of 12-week-old *Angpt1*ΔNC mice with control littermates, and total RNA was collected from the limbus/iridocorneal angle region and the retina. RNA was also purified from the lung as a control tissue not derived from the neural crest. Quantitative, real-time PCR revealed almost complete ablation of *Angpt1* expression in the limbal/iridocorneal angle region of *Angpt1*ΔNC eyes, confirming the neural crest origin of *Angpt1* expressing cells in the iridocorneal angle (Fig. 1D). Only a minor reduction in *Angpt1*expression was observed in the retina, consistent with previous data suggesting that in addition to retinal pericytes, *Angpt1* is expressed in neuroepithelium-derived amacrine cells^25,32^. As expected, no reduction in *Angpt1* expression was observed in the lung. Interestingly, we noted that compared to the iridocorneal angle region, retinal *Angpt1* expression was very low in wild-type mice (Supplementary Fig. 1), consistent with previous findings that *Angpt1* is not required for retinal angiogenesis despite the well-described reliance on TEK and ANGPT2 signaling.

### Angpt1ΔNC mice are a model of developmental glaucoma

**Fig. 2:**
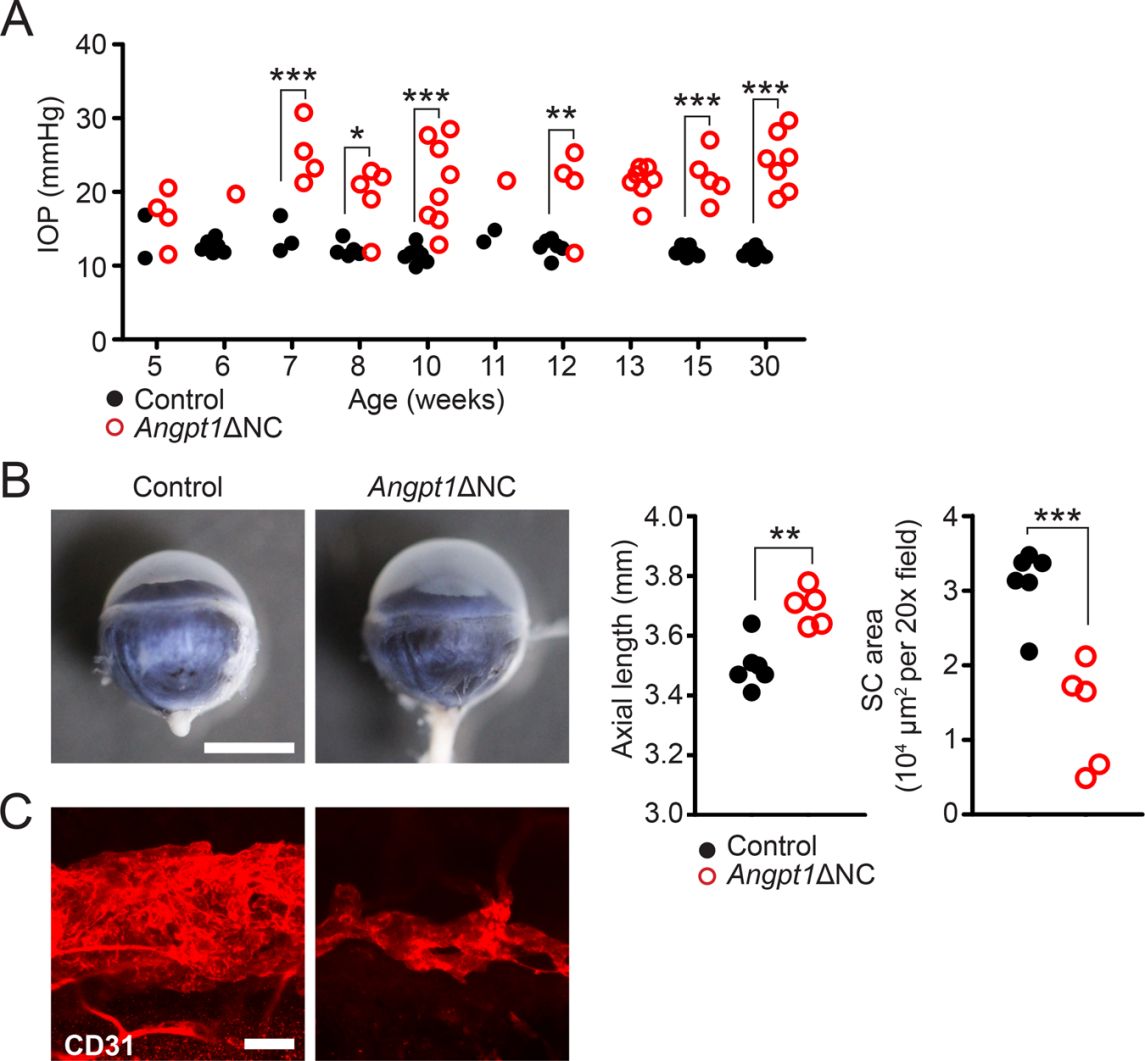
*Angpt1*ΔNC mice exhibit ocular hypertension and hypomorphic Schlemm’s canal development. **A** Compared to control littermates, rebound tonometry revealed elevated intraocular pressure in *Angpt1*ΔNC mice beginning at 7 weeks of age. Each group was measured at only a single timepoint, except for one group measured at 10 and 30 weeks of age. Each datapoint represents the average of left and right eyes from a single animal. Genotype *p* < 0.0001 as determined by two-way ANOVA, Df_genotype_ = 1, F_genotype_ = 95.67. *p ≤ 0.05, **p ≤ 0.01, ***p ≤ 0.001 as determined by Bonferroni posttests. Lacking littermate controls, 13-week timepoint was excluded from statistical analysis. *n* = 36 (control) and 45 (*Angpt1*ΔNC) animals from eight independent litters. **B** By 15 weeks, increased axial length was apparent in enucleated eye globes of *Angpt1*ΔNC mice. *n* = 6 (control), 5 (*Angpt1*ΔNC), *p* = 0.0013. **C** Compared to littermate controls, whole-mount confocal microscopy using anti-CD31 antibody revealed reduced Schlemm’s canal area in *Angpt1*ΔNC mice, suggesting that ocular hypertension was due to insufficient aqueous humor outflow. *n* = 6 (control), 5 (*Angpt1*ΔNC), *p* = 0.0008. **p ≤ 0.01, ***p ≤ 0.001 as determined by two-tailed Student’s *t*-test. Df = 9. Scale bars represent 2 mm (**B**) and 50 μm (**C**). 20× fields used for quantification in (**B**) represent an area of 65,025 μm^2^.

We next examined *Angpt1*ΔNC mice for signs of glaucoma, to determine if neural crest specific *Angpt1* deletion was sufficient to recapitulate the phenotype of inducible whole-body knockouts. Using rebound tonometry, elevated IOP was detected in *Angpt1*ΔNC mice by 7 weeks of age (Fig. 2A). IOP remained elevated throughout life, with increased axial length (a surrogate marker for globe expansion) observed in *Angpt1*ΔNC eyes by 15 weeks of age (Fig. 2B, Control: 3.5 ± 0.03 mm, *Angpt1*ΔNC: 3.67 ± 0.03 mm, p = 0.0013). CD31-positive SC area was also dramatically reduced (Fig. 2C, Control: 3.11 ± 0.19, *Angpt1*ΔNC: 1.33 ± 0.32 10^4^ μm^2^/20x field, *p* = 0.0008) suggesting ANGPT1 secretion by TM cells of the neural crest lineage is essential for proper canal formation and IOP homeostasis.

**Fig. 3:**
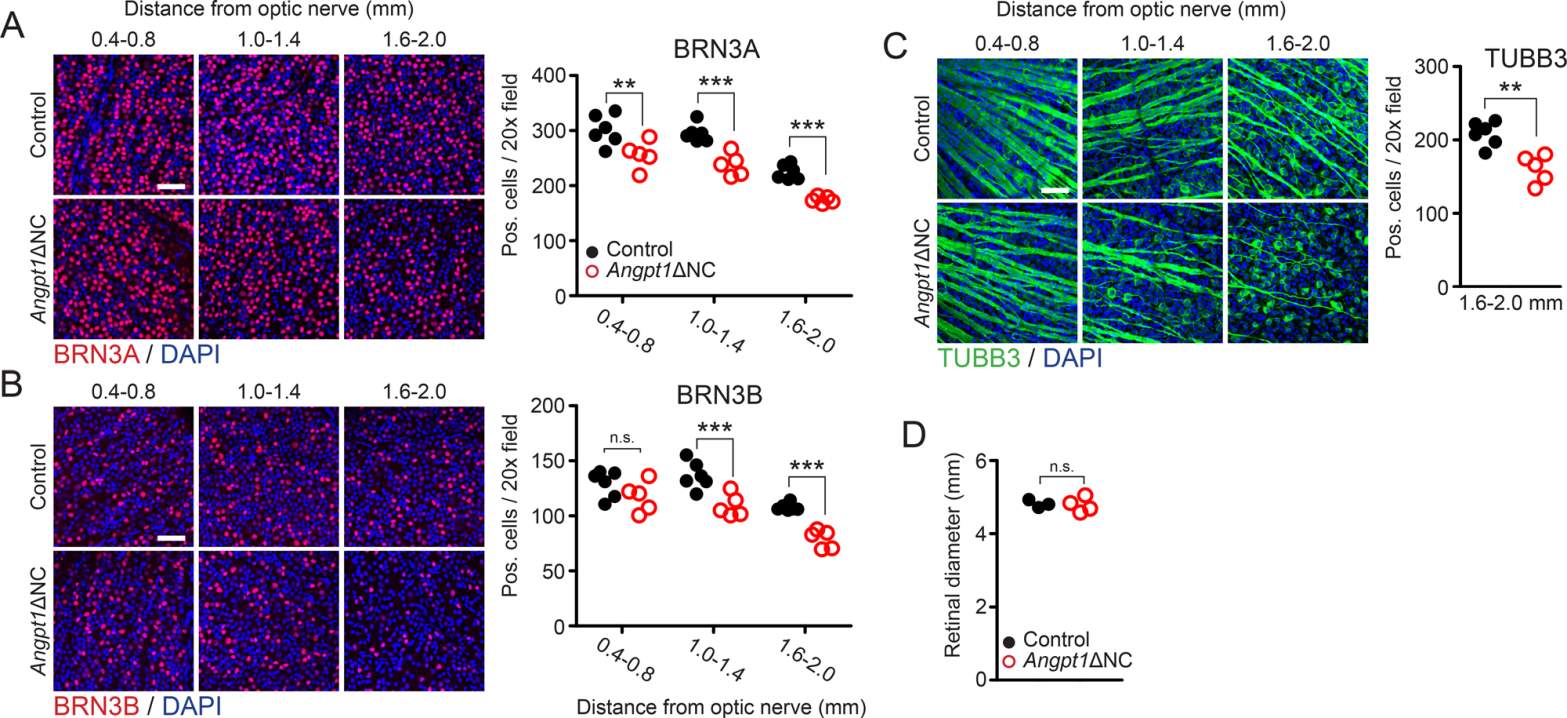
Neural crest specific *Angpt1* knockout mice exhibit characteristics of glaucomatous neuropathy. Compared to control littermates, retinal flat mounts from 15-week-old *Angpt1*ΔNC mice showed reduced numbers of (**A**) BRN3A and (**B**) BRN3B positive ganglion cell nuclei. This reduction was especially pronounced in the peripheral retina. **C** A similar reduction in retinal ganglion cells was observed in *Angpt1*ΔNC mice stained with anti-TUBB3 antibody. Due to visual obstruction by TUBB3-positive nerve fibers in the central retina, TUBB3-positive cells were quantified only in the periphery. *p* = 0.0016. **D** Retinal diameter was unchanged in *Angpt1*ΔNC mice at 15 weeks. Each data point represents average values obtained from a single animal. n.s. *p* > 0.05. **p ≤ 0.01, ***p ≤ 0.001 as determined by two-way ANOVA followed by Bonferroni’s correction (**A**, **B**) or two-tailed Student’s t-test, Df = 9 (**C**, **D**). *n* = 6 (control) and 5 (*Angpt1*ΔNC). Two-way ANOVA Df_genotype_ = 1, *F*_genotype_ = 55.35 (**A**), 37.27 (**B**). Scale bars represent 50 μm. 20× fields used for counting comprise an area of 65,025 μm^2^.

Vision loss in high-pressure glaucoma is due to IOP-induced apoptosis of retinal ganglion cells (RGCs). To further characterize the glaucoma phenotype in *Angpt1*ΔNC mice, retinal flat mounts were prepared and RGCs were counted by confocal microscopy. While neonatal mice exhibited no differences in number of BRN3A (*Pou4f1*) or BRN3B (*Pou4f2*)-positive RGCs at postnatal day 5 (Supplementary Fig. 2), by 15 weeks a marked reduction in BRN3-positive RGCs was observed in *Angpt1*ΔNC mice (Fig. 3A, B). This RGC loss was especially pronounced in the peripheral retina (Loss of BRN3A + cells: central: 15.1%, mid 19.1%, peripheral: 22.4%; BRN3B: central: 9%, mid: 21.1%, peripheral: 26.6%), consistent with our previous observations in whole-body *Angpt1* knockout mice and with other rodent models of IOP-induced glaucomatous neuropathy. As some studies have reported that BRN3 expression is lost in injured RGCs before cell death^33^, we validated RGC loss by staining for β-III-Tubulin (TUBB3), a universal marker of RGCs (Fig. 3C). TUBB3 positive nerve fibers obstructed cell counting in the central retina, but peripheral TUBB3-positive RGC loss was consistent with that observed by BRN3 staining (TUBB3: 23.1%, BRN3A + BRN3B: 23.8%). We did not observe increased retinal diameter in these animals (Fig. 3D), supporting the hypothesis that reduced RGC density was due to cell loss rather than retina deformation.

We next examined the retinal vasculature to confirm that RGC loss in *Angpt1*ΔNC mice was due to IOP elevation and not retinal ischemia. At postnatal day 5, when the superficial retinal vasculature is undergoing rapid angiogenesis, we observed normal CD31-positive vascular patterning in *Angpt1*ΔNC retinas with no difference in sprouting progression at either the venous or arterial sprouting fronts (Supplementary Fig. 3A). The retinal vasculature was also normal at 15 weeks, when we observed marked loss of RGCs (Fig. 3B), with no apparent differences in patterning or area of the superficial, intermediate, or deep vascular layers (Supplementary Fig. 3B, C).

### Identification of Svep1: a new gene for PCG

Recent findings have solidified our understanding of SC as a unique “hybrid” endothelium with aspects of both blood and lymphatic vascular identity and have highlighted the importance of lymphatic signaling molecules such as PROX1 and FLT4 in canal regulation^5,34^. In a further example of the similarity between lymphatic and SC biology, a likely loss-of-function variant in *SVEP1*, encoding an extracellular matrix protein required for lymphatic development and valve formation, was recently identified as a modifier of PCG disease in a large family with five affected generations^22^. In addition, a common *SVEP1* variant (rs61751937) has been identified via GWAS as a risk allele for POAG, further supporting a role for *SVEP1* in IOP homeostasis^23^. In the lymphatic vasculature, SVEP1 is expressed by lymphatic-associated mesenchymal cells, and as the SVEP1 receptor integrin α9β1 is strongly expressed in the SC endothelium^34,35^ we hypothesized that SVEP1- integrin α9β1 signaling may be another example of TM-SC crosstalk.

To examine the role of SVEP1 in SC development and function, we obtained frozen sperm from the European Conditional Mouse Mutagenesis Program (EUCOMM) from mice carrying a *Svep1*-floxed allele (Svep1^tm1b(EUCOMM)Hmgu^, Supp. Fig. 4A). After breeding with a FLPe-recombinase expressing mouse to remove the neomycin selection cassette used in targeting, *Svep1*-floxed mice were crossed with mice carrying Cre-recombinase (Tg(EIIa-cre)C5379Lmgd) to generate a *Svep1*^null^ allele. As previously reported, *Svep1*^null/null^ mice were not viable and no living pups were recovered after birth^20^. Confirming these findings and the functionality of our *Svep1* knockout mice, living *Svep1*^null/null^ embryos recovered by cesarean section at embryonic day (E) 18.5 had marked subcutaneous edema (Supp. Fig. 4B). Lymphatic vessels in the dorsal skin were dilated and disorganized, lacking lymphatic valves and sprouting tips (Supp. Fig. 4C). Lymphatic vessels were present in the mesentery, but valves were absent suggesting failed collecting vessel development as previously reported (Supp. Fig. 4D).

**Fig. 4:**
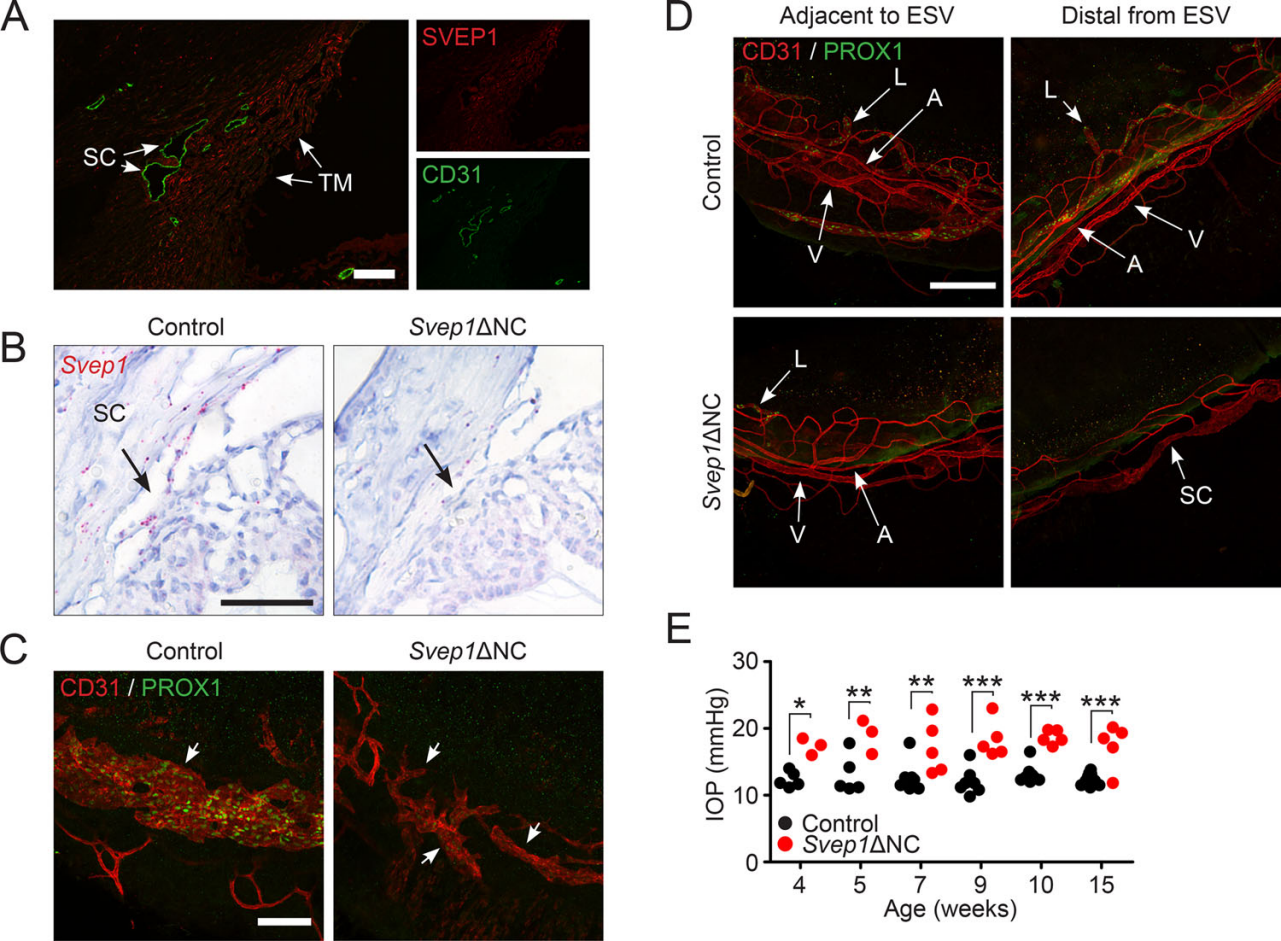
Neural-crest derived SVEP1 is essential for Schlemm’s canal formation. **A** SVEP1 expression was observed in the trabecular meshwork (TM) but not Schlemm’s canal (SC) endothelium following immunostaining of the human iridocorneal angle. **B** Loss of *Svep1* expression in *Svep1*ΔNC mice was confirmed by in situ hybridization. Confocal microscopy at postnatal day 10 revealed formation of a hypomorphic Schlemm’s canal with no apparent PROX1 expression in *Svep1*ΔNC eyes. **D** In addition to defects in Schlemm’s canal formation, defects in the limbal vascular and lymphatic networks were discovered in *Svep1*ΔNC mice. In contrast to the organized pattern formed by the perilimbal vein (V) and circular limbal artery (A) found in control mice, these vessels were disorganized in *Svep1* mutants, with sparse lymphatic vessels (L) and loss of venous/arterial patterning in regions distal to the episcleral vein (ESV). Confirming that this hypomorphic SC and disorganized distal outflow pathway provided insufficient drainage function, rebound tonometry revealed elevated intraocular pressure beginning at 4 weeks of age. **E** *n* = 15 (control) and 8 (*Svep1*ΔNC) animals from two independent litters. *p ≤ 0.05, **p ≤ 0.01, ***p ≤ 0.001 as determined by two-way ANOVA followed by Bonferroni’s correction. Df_genotype_ = 1, *F*_genotype_ = 99.2. Scale bars represent 50 μm (**B**), 100 μm (**A**, **C**) and 250 μm (**D**).

As SVEP1 is robustly expressed by the TM in human eyes (Fig. 4A), we used the same neural crest deletion approach described above to obtain adult mice lacking SVEP1 in uveal tissues (*Svep1*ΔNC mice). *Svep1*ΔNC mice were born normally with no apparent phenotype. However, paraffin sections revealed a hypomorphic SC phenotype when *Svep1* deletion was validated by in situ hybridization (Fig. 4B). This finding was confirmed by confocal whole-mount imaging of the limbal region, where *Svep1*ΔNC mice exhibited a severely hypomorphic SC lacking PROX1 expression (Fig. 4C). In addition to these SC defects, the limbal vasculature of *Svep1*ΔNC mice was disorganized (Fig. 4D). This phenotype was especially pronounced distal to the episcleral vein, where the normal patterning of the perilimbal vein and circular limbal artery was lost and replaced with a network of irregular capillaries. In addition, in mutant eyes lymphatic vessels were observed only adjacent to the episcleral vein in regions where normal vascular patterning was partially retained.

Rebound tonometry revealed elevated IOP in *Svep1*ΔNC mice beginning at 4 weeks of age, consistent with a PCG-like phenotype and confirming that uveal SVEP1 is essential for IOP homeostasis (Fig. 4E).

### Understanding the cellular basis of neural crest-SC crosstalk

The importance of TM-SC signaling via ANGPT1 and SVEP1 for SC development and IOP homeostasis is reminiscent of other, well-characterized, endothelial/mural cell interactions such as vascular endothelial cells with adjacent pericytes or glomerular endothelium with the renal mesangium^36^. These data also suggest that similar crosstalk via additional pathways might also be important in outflow regulation and could provide new targets for glaucoma therapy. To investigate this possibility, we used a single-cell transcriptomics approach to construct a cellular atlas of the iridocorneal angle region, using known morphological changes in *Angpt1*ΔNC eyes as a template to guide cell cluster identification.

**Fig. 5:**
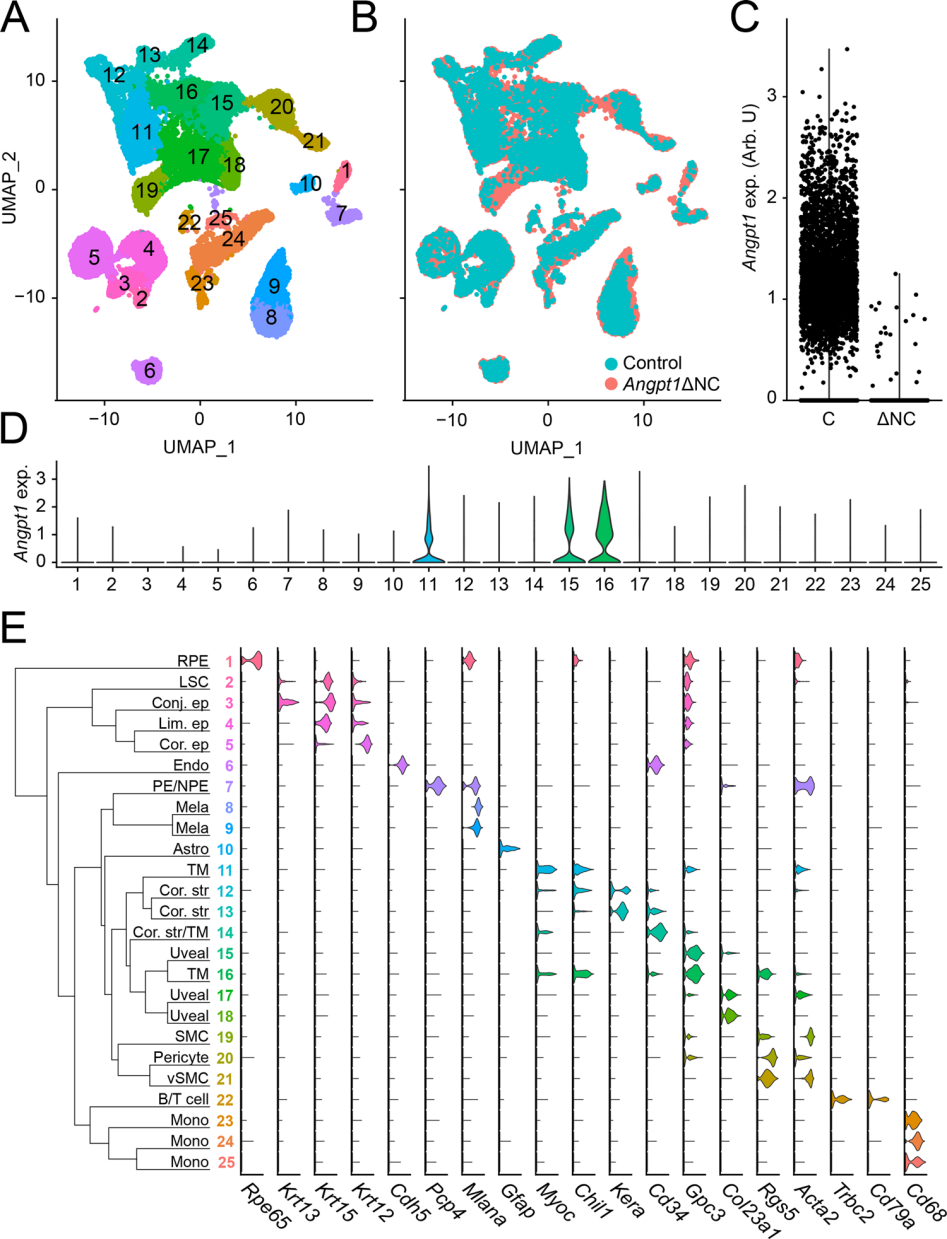
Single-cell RNA sequencing of the iridocorneal angle region of control and glaucomatous *Angpt1*ΔNC eyes. **A** Single-cell RNA sequencing was performed on pooled eyes from 6-week-old control and *Angpt1*ΔNC mice. Samples were integrated using CCA and clustered using the Seurat package in R before plotting using UMAP. **B** Overlaying samples from control and *Angpt1*ΔNC eyes shows that similar cell populations were present in each sample. **C** *Angpt1* was detected in only a small number of cells isolated from *Angpt1*ΔNC eyes, indicating that the majority of *Angpt1*-expressing cells are derived from the neural crest. **D** *Angpt1* expressing cells in WT mice were concentrated in clusters 11, 15, and 16. **E** Dendrogram and representative marker gene expression in each cluster identified. RPE, retinal pigment epithelium, LSC, limbal stem cell, conj. ep, conjunctival epithelium, lim. ep., limbal epithelium, cor. ep., corneal epithelium, mela, melanocyte, cor. str., corneal stroma, TM, trabecular meshwork, PE/NPE, ciliary (non) pigmented epithelium.

Enucleated eyes from 2 to 3 mixed-sex 6-week-old *Angpt1*ΔNC or control mice were pooled and much of the conjunctiva was removed before microdissection. Globes were bisected, the retina and iris were removed and the iridocorneal angle/corneal limbal region was isolated by cutting the sclera immediately posterior to the ciliary body and cornea anterior to the TM. A single-cell suspension was prepared, and barcoded libraries were generated using the 10x Genomics Chromium platform. Following sequencing and quality control filtering, a total of 19,236 control and 6772 *Angpt1*ΔNC cells were included in our analysis (Supp. Fig. 5). After dataset integration using an anchor-based CCA pipeline implemented in Seurat^37^, initial clustering performed at a low resolution revealed 25 distinct cell groups, ranging in size from 166 (cluster 2) to 2287 (cluster 17) cells (Fig. 5A). All groups contained cells from both control and *Angpt1*ΔNC samples, although cluster 1, enriched in cells strongly expressing RPE markers was nearly absent in the *Angpt1*ΔNC sample—likely due to differences in dissection when removing the retina (Fig. 5B). Very few *Angpt1* expressing cells were detected in the *Angpt1*ΔNC population (Fig. 5C, Control: 17.26%, *Angpt1*ΔNC: 0.34%). The majority of these remaining *Angpt1*-expressing cells were present in clusters with high numbers of *Angpt1*-expressing cells in control eyes, suggesting the apparent *Angpt1*expression in these cells was not due to erroneous sequencing reads but to cells which had escaped *Wnt1*-cre mediated gene deletion or nonsense-mediated decay following excision of exon 1, (Supplementary Fig. 6). These results confirmed that *Angpt1*-expressing cells in the iridocorneal angle/limbal region are derived from the neural crest.

### Cluster classification

Our initial clustering of limbal/iridocorneal angle cells differentiated several clusters of cells characterized by unique transcripts which were readily identified as RPE (cluster 1, Fig. 5A, E, supplemental dataset 1), conjunctival (3), limbal (4), and corneal (5) epithelium, limbal stem cells (2), endothelial cells (6), ciliary epithelia (7), melanocytes (8 & 9), astrocytes (10), corneal stromal keratinocytes (12 & 13) smooth muscle (19), pericytes (20), vascular smooth muscle (21), B and T cells (22), and monocytes (23–25). In addition to these clearly recognizable populations, our clustering identified several groups with more ambiguous expression profiles. Clusters 15–17 contained stromal cells. High levels of *Angpt1* expression were observed in clusters 15 and 16, suggesting that these may contain the uveal *Angpt1*-expressing stroma population observed histologically (Figs. 1A, 5D). Known markers of TM cells including *Chil1* and *Myoc* were also detected in some cells of cluster 16. Cluster 14 largely consisted of *Cd34*-positive cells, which shared many markers with fibroblasts, including *Mfap5*, *Clec3b* and *Tnxb*, suggesting that these may represent the *Cd34*-positive corneal stroma population which has been previously described^38^. However, a subset of cells in cluster 14 also expressed *Myoc* and *Chil1*. Cluster 11 contained *Cdh2*-positive cells hypothesized to be corneal endothelium as well as *Acta2*-positive cells expressing *Angpt1* and the TM markers *Chil1* and *Myoc*. The distribution of putative TM cells between clusters 11 (corneal endothelium-like), 14 (corneal stroma/fibroblast) and 15/16 (uveal stroma) is consistent with previous studies identifying multiple cell types in the TM, including fibroblast-like “beams” and pericyte-like juxtacanalicular (JCT) or trabecular cells^39^.

### Identification of SC endothelial cells

**Fig. 6:**
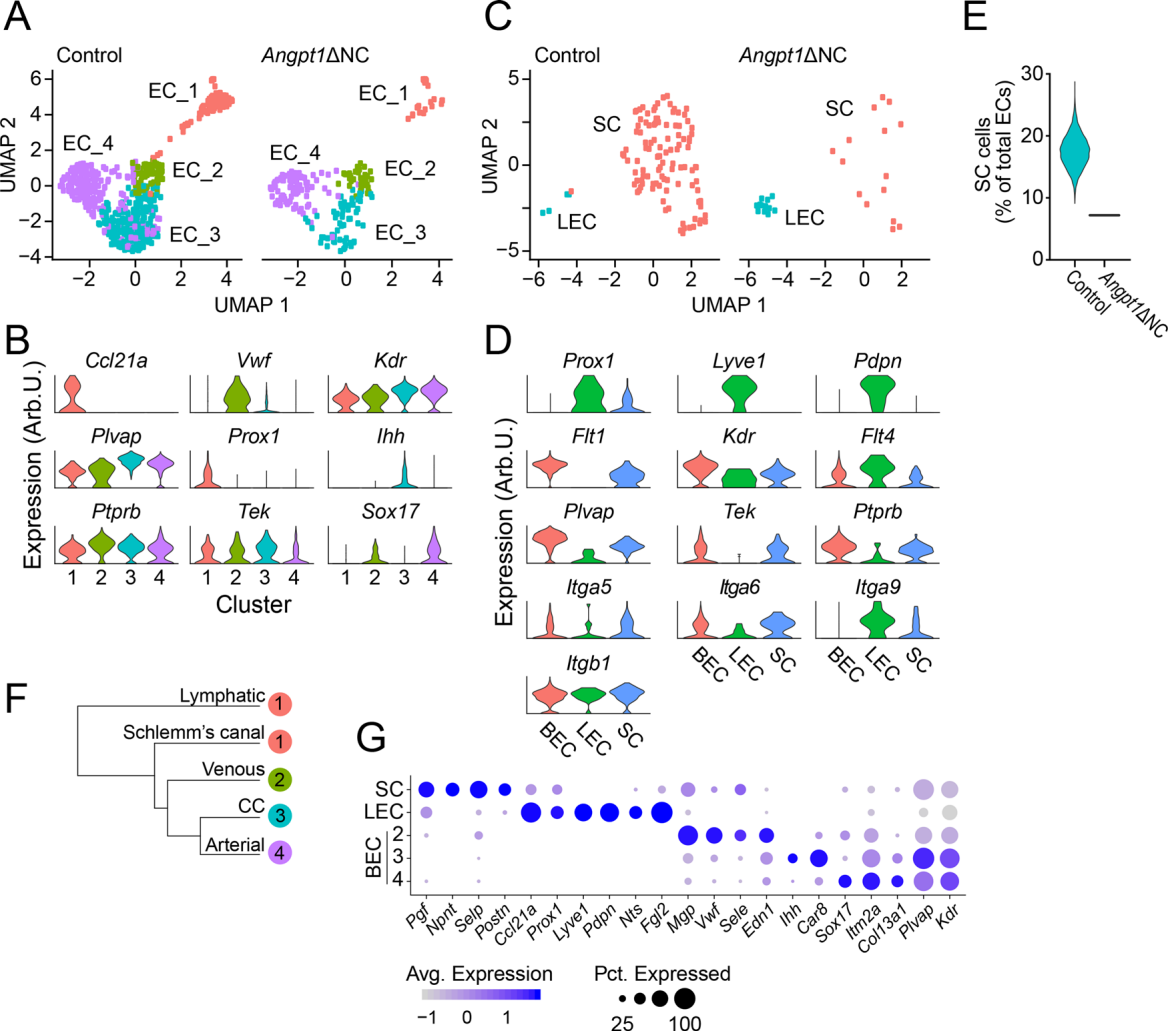
Single-cell RNA sequencing identified Schlemm’s canal endothelial cells. **A** *Cdh5* + endothelial cells were identified from the total cell population and reclustered using a new set of principal components, resulting in four endothelial cell clusters. **B** Expression analysis of marker genes present in the four unique endothelial populations. Examination of the endothelial populations in (**A**) revealed that *Ccl21a*-positive cells in cluster 1 were comprised of two distinct populations. One of these populations was nearly absent in *Angpt1*ΔNC eyes, suggesting that they might represent SC endothelial cells. **C** We, therefore, isolated these cells and reclustered them, allowing detailed analysis. **D** Expression analysis revealed that the presumptive lymphatic endothelial cells (LECs) expressed classic lymphatic markers such as *Prox1*, *Pdpn,* and *Lyve1*. In contrast, Schlemm’s canal endothelial cells (SCs) expressed *Plvap*, *Tek* and *Ptprb* in addition to *Flt1* while retaining low-level expression of *Prox1* and *Flt4*. **E** Consistent with our histological results, very few SC endothelial cells were identified in Angpt1ΔNC mice. To exclude the possibility that was due to sampling variability, we performed bootstrapping analysis, picking a sample of endothelial cells of equal size to our knockout sample from the WT dataset. 10,000 iterations were performed, revealing that *Angpt1* knockout mice have far fewer SC endothelial cells then controls. Distribution of simulated samples is shown as a violin plot compared to the true proportion of SC endothelial cells identified in *Angpt1* knockout eyes. **F** Dendrogram illustrating the transcriptomic relationship between endothelial cell clusters identified. **G** Identification of unique markers of Schlemm’s canal (SC), ocular lymphatic (LEC) and blood vascular (BEC) endothelium.

While recent immunostaining studies have provided important insights into SC biology, the difficulty isolating pure populations of SC endothelial cells for cell culture or transcriptional profiling has limited our understanding of TM-SC crosstalk and the relationship between SC, blood vascular and lymphatic gene expression pattern and function. As our histological analysis of *Angpt1*ΔNC eyes revealed a marked reduction in SC size and SC endothelial cell numbers, we reasoned that very few of these cells would be present in our *Angpt1*ΔNC samples, providing a tool for distinguishing populations. To identify SC cells, we isolated *Cdh5*-expressing cluster 6 from our dataset, and reclustered these cells using a new set of principal components (Fig. 6A). Four distinct endothelial clusters were identified, which we designated EC1-4. EC2-4 appeared to contain blood vascular endothelial cells, while EC1 contained *Prox1* positive cells expressing high levels of *Ccl21a*, suggesting a lymphatic-like phenotype (Fig. 6B). EC3 expressed high levels of *Kdr*, *Plvap* and *Ihh*, a newly described marker of the choriocapillaris^40^, suggesting that these were cells from the peripheral choriocapillaris while EC2 and 4 displayed venous and arterial phenotypes, respectively. Intriguingly, our UMAP projection of the lymphatic-like cells in cluster 1 delineated two distinct groups, the larger of which contained far fewer cells in *Angpt1*ΔNC mice—suggesting that this group might represent SC endothelial cells. Accordingly, we isolated cluster 1 from the remaining endothelial cells and reclustered them using a third set of principal components, revealing two distinct cell populations (Fig. 6C). Consistent with previous reports of SC gene expression, the larger population lacked *Lyve1* and *Pdpn* expression while expressing low levels of *Prox1*, suggesting a SC identity^5^. The smaller cluster contained cells expressing higher levels of *Prox1* as well as *Lyve1* and *Pdpn*, an expression pattern typical of lymphatic endothelial cells (LECs, Fig. 6D, Supp. Fig. 7). Putative SC endothelial cells expressed a combination of blood and lymphatic endothelial integrins, sharing expression of *Itga5*and *Itga6* with blood endothelial cells and the SVEP1 receptor *Itga9* with lymphatics. Compared to the control samples, very few putative SC endothelial cells were observed in the *Angpt1*ΔNC dataset, providing further evidence that this cluster originated from SC (Fig. 6E, Control: 17.5% of total ECs, *Angpt1*ΔNC: 7.2%). Consistent with the origin of SC sprouting from blood-filled capillaries^4^, the transcriptome of SC endothelial cells was more closely related to blood vascular endothelial cells than to LECs (Fig. 6F), although they were differentiated by several highly expressed markers including *Ccl21a*, *Prox1*, *Nupr1* and *Pgf* (Fig. 6G, full data in supplemental dataset 1). Compared to LECs, SC endothelial cells lacked classical LEC markers such as *Pdpn* and *Lyve1*, with increased expression of *Cxcl10* and *Serpine1*.

### Identification of TM cell clusters

**Fig. 7:**
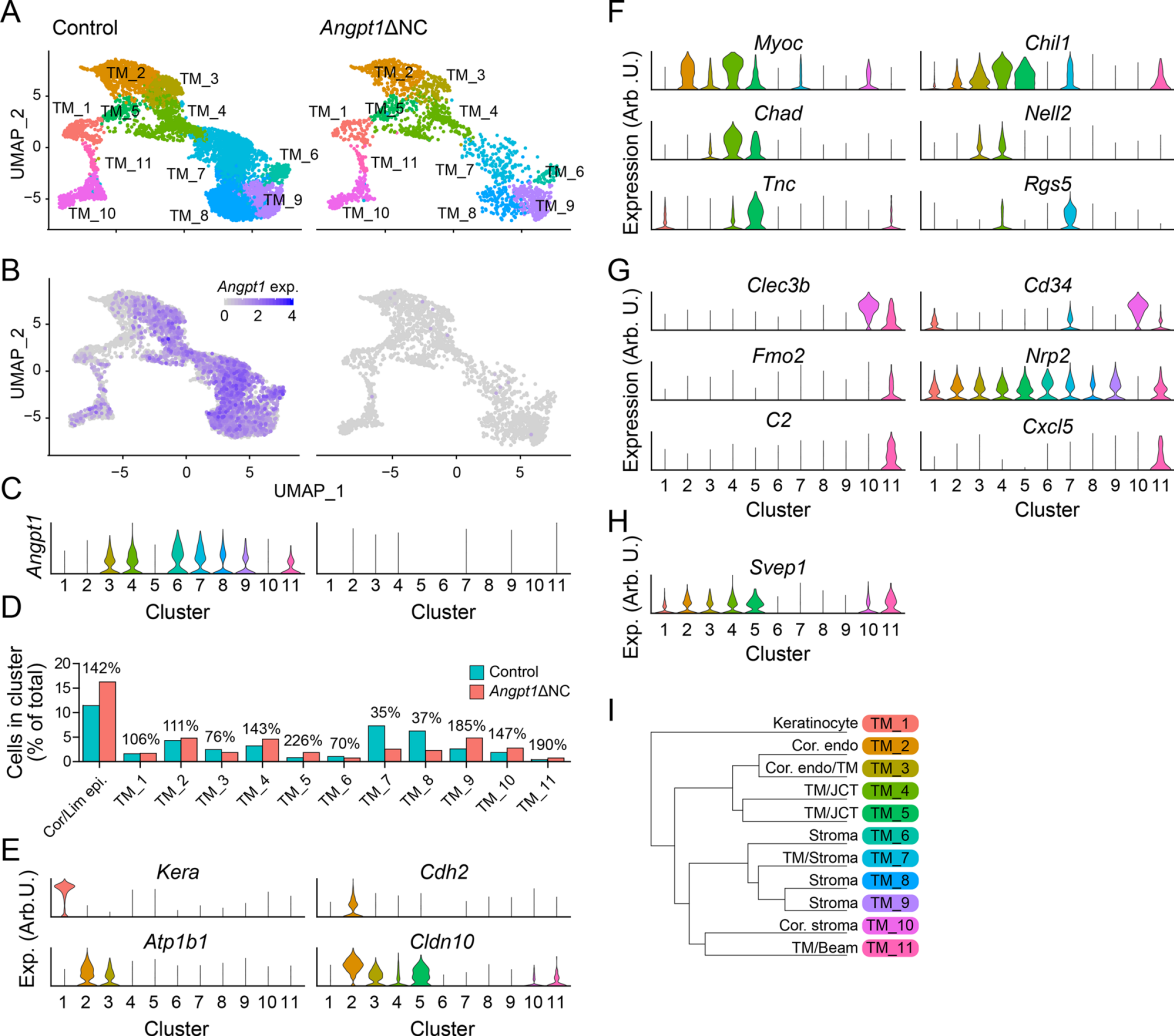
Analysis of putative trabecular meshwork cells. **A** Clusters containing cells expressing known TM markers were isolated from the full dataset and reclustered in a new set of principal components. **B**, **C** *Angpt1* expression in putative TM cell clusters from control and *Angpt1*ΔNC eyes. **D** Bars indicate cell cluster size as a percentage of total sequenced cells of each genotype. Noted percentages indicate relative abundance of each cluster in *Angpt1*ΔNC eyes compared to control sample. **E**–**G** Marker gene analysis in wild-type samples was used for cluster identification. E Cells in TM1 revealed features of keratinocytes while TM2 contained corneal endothelia. **F** TM4 and 5 exhibit an expression pattern consistent with trabecular meshwork or JCT cells. **G** TM10 contained Cd34+ cells which appeared to be corneal stromal cells. The expression pattern in TM11 was consistent with trabecular meshwork fibroblast like “beam” cells. (**H**) *Svep1* expression in TM cell clusters. (I) Dendrogram highlighting the relationship between identified cell clusters.

Our preliminary analysis showed that cells expressing known TM marker genes such as *Myoc* and *Chil1* were included in clusters 11, 12, 14, and 16 (Fig. 5A, D), although they did not comprise a majority of cells in any of these groups—indicating our initial clustering was too low-resolution to differentiate these phenotypes from the closely related cells in their overall clusters. Accordingly, to identify individual TM cell populations, we isolated these groups along with the closely related cluster 15 and reclustered them using a third set of principal components. Eleven groups were identified, which we designated TM1-11 (Fig. 7A). *Angpt1* expressing cells were present in clusters TM3, 4, 6, 7, 8, 9, and 11 (Fig. 7B, C) although were most represented in clusters TM4, 6 and 7. As expected, few or no *Angpt1*-positive cells were observed in *Angpt1*ΔNC samples. All clusters were present in both control and *Angpt1*ΔNC samples, although the *Angpt1*ΔNC sample contained proportionally fewer cells in the clusters identified as uveal stroma, and more cells in the clusters originating from the cornea (Fig. 7D). It is possible that this reduction in uveal clusters is indicative of the hypomorphic TM we have previously observed in the eyes of *Angpt1* and *Tek* knockout mice. However, we also observed proportionally greater numbers of corneal epithelial cells in the *Angpt1*ΔNC sample, so we cannot exclude the possibility that this difference was due to variability in dissection. Small differences in the location where cornea and sclera were cut would likely result in shifts in representation of these populations within our sample.

After reclustering, differential gene expression analysis revealed that TM1 contained keratinocytes, while TM2 contained cells expressing markers of the corneal endothelium, including *Cdh2*, *Atp1b1,* and *Cldn10* (Fig. 7E). Cells in TM4 and 5 expressed TM markers including *Myoc*, *Chil1*, *Chad*, *Nell2* as well as *Angpt1* (Fig. 7F). The gene encoding Tenascin C, a predominantly JCT-expressed protein upregulated in regions of high outflow^41^, was expressed in both TM4 and 5, although expression was highest in TM5. In contrast, expression of *Rgs5*, a pericyte marker also expressed in TM^42,43^, was present in TM4 but not TM5.

Clusters TM10 and 11 contained cells expressing fibroblast markers including *Clec3b* (Fig. 7G). In addition, *Myoc* expression was detected in TM10, suggesting that these cells may be analogous to the”Beam A” cluster described by van Zyl et al.^39^. However, we found that these cells also expressed *Cd34*, which is absent from the TM^44,45^. Instead, we hypothesize that TM10 may represent a *Cd34*-postive corneal stromal population which has been previously described^38,46,47^. *Cd34* expression was not present in closely related TM11, which contained *Chil1*-positive cells also expressing *Fmo2*, suggesting that they were analogous to the “Beam X” population described by van Zyl et al.^39^. Unlike TM10, this cluster also expressed *Nrp2*, which is strongly expressed throughout the TM (Fig. 7G, Supp. Fig. 8). TM11 also expressed complement-related genes, including *C2* and *Cfb* as well as chemokines including *Cxcl5*. TM6-9 contained *Angpt1*, *Pdgfrb*-positive cells which we interpreted to be the uveal *Angpt1*-GFP-positive cells we observed histologically (Fig. 1A). Interestingly, TM7 strongly expressed *Rgs5* as well as *Chil1*, suggesting that this cluster may be associated with the TM. Consistent with the results of our histological and tissue-specific knockout studies, *Svep1* was strongly expressed in putative TM/JCT clusters TM4 and TM5 (Fig. 7H). A dendrogram highlighting the relationship between clusters is shown in Fig. 7I.

### Expression of POAG and IOP-related genes and pathways

Having identified a population of putative SC endothelial and TM cells, we set out to identify genes of interest for future glaucoma studies. A list of SC and TM-expressed non-mitochondrial genes with normalized expression levels >0.3 was identified (Supplementary dataset 2). This list was then compared to a curated list of genes associated with PCG, POAG, IOP elevation, or SC function and development via GWAS, genetic studies, or experimental models (Supplementary Table 1). Twenty-one genes were identified in SC endothelial cells, including *Cav1*, *Cav2*, *Tgfbr3*, *Foxc1*, *Tek*, *Flt1*, *Kdr*, and *Flt4* (Table 1). Thirty genes were identified in putative TM cell clusters, including *Foxc1*, *Myoc*, *Angpt1, Svep1* and *Vegfa*. Some genes with known expression in either SC or TM were detected above the threshold in both cell populations, including *Cyp1b1*, *Vegfa* and *Cav1*. However, their expression was heavily skewed, with expression of *Cyp1b1* and *Vegfa* higher in TM clusters (*Cyp1b1*: Avg. expression: TM: 2.44; SC: 0.83; *Vegfa*: Avg. expression: TM: 3.29; SC: 0.75). *Cav1* expression was more highly expressed in SC endothelium (Avg. exp: TM: 0.37, SC: 2.84).

**Table 1:** Glaucoma and IOP-associated genes expressed in the TM and Schlemm’s canal.

SC	TM	Shared genes
*Tek	*Myoc	*Foxc1
***Kdr***	*Svep1	*Cyp1b1
***Flt1***	*Angpt1	***Vegfa***
***Bmp4***	*Tgfb2*	*Abca1*
***Flt4***	*Bicc1*	*Atxn2*
*Tgfbr3*	*Col6a2*	*Cav1*
*Gas7*	*Dcn*	*Fermt2*
*Cav2*	*Vegfc*	*Arhgef12*
*Cttnbp2*	*Cdh11*	*Stag1*
	*Loxl2*	*Tmco1*
	*Loxl1*	*Ets1*
	*Col12a1*	*Fmnl2*
	*Efemp1*	
	*Adamts2*	
	*Gmds*	
	*Fbn1*	
	*Thbs2*	
	*Ltbp1*	

Genes with average per-cell normalized expression over 0.3 arbitrary units in the specified cluster were compared to a curated list of glaucoma, IOP or SC-associated loci identified by GWAS, genetic studies or experimental models. Genes with known functional links to glaucoma in animal models are identified by bold text and validated causative genes for human disease are marked with an asterisk (*).

### TM-SC crosstalk and the ANGPT-TEK axis are promising therapeutic targets for SC-directed glaucoma therapy

The essential nature of ANGPT-TEK signaling in SC development led us to hypothesize that this pathway might be a valuable target for glaucoma therapy aimed at increasing SC function and outflow through the conventional route. We have recently developed a highly effective ANGPT1-mimetic fusion protein in which the ECM-binding and oligomerization domains of ANGPT1 were replaced with a heptameric scaffold derived from the C-terminus of serum complement protein C4-binding protein α. The resulting fusion protein is soluble in aqueous solutions at physiological pH and forms stable heptamers which robustly activate TEK in vivo and in vitro^48^.

**Fig. 8:**
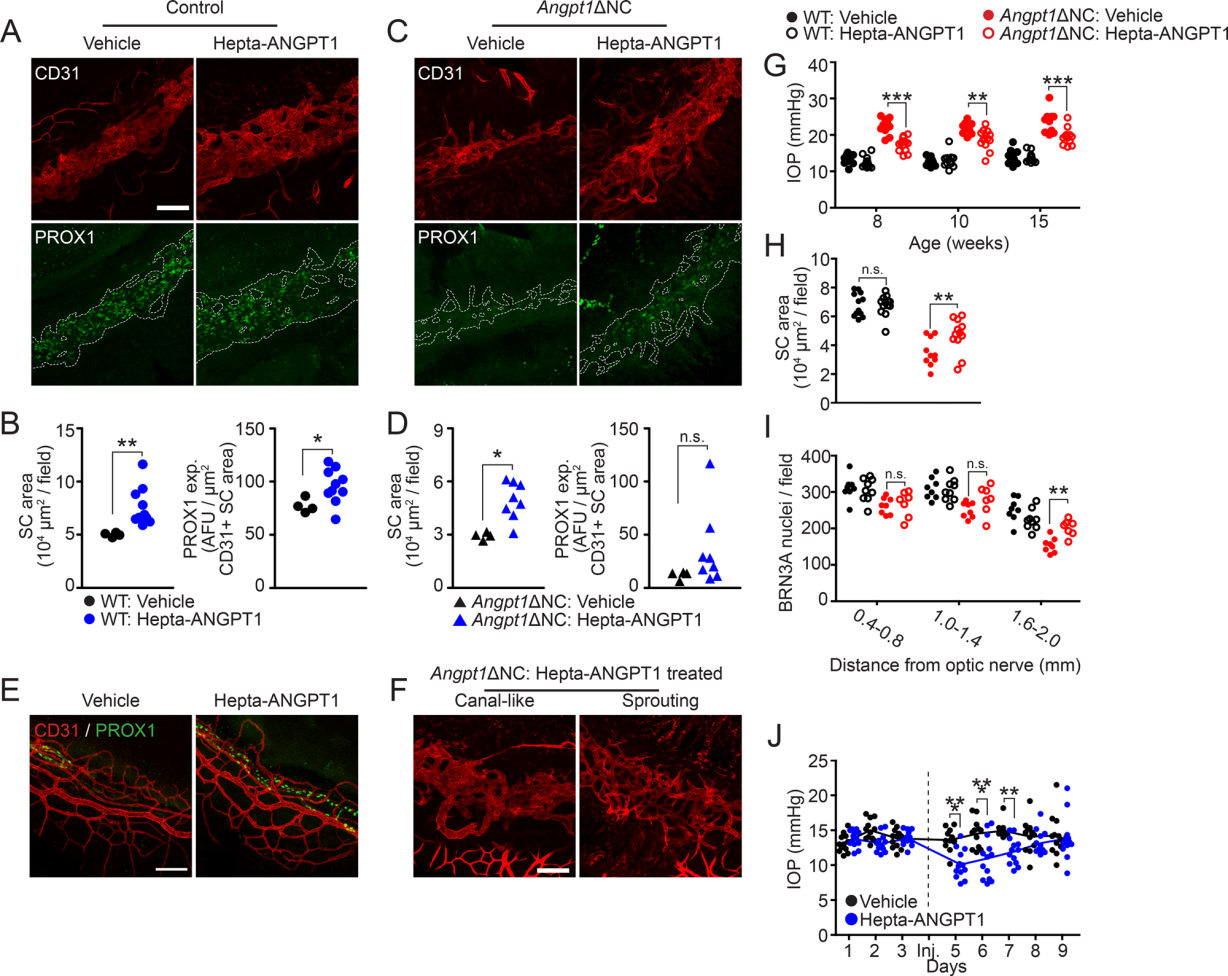
ANGPT1 mimetic treatment increased SC area and lowered IOP in mice. (**A**, quantified in **B**) Intra-peritoneal (IP) treatment with Hepta-ANGPT1 from birth to P14 led to increased SC area in WT (*p* = 0.0014) and (**C**, quantified in **D**) *Angpt1*ΔNC eyes (*p* = 0.0008). Wildtype, *n* = 4 (vehicle) and 10 (Hepta-ANGPT1), *Angpt1*ΔNC, *n* = 4 (vehicle) and 8 (Hepta-ANGPT1) animals from four independent litters. PROX1 expression in WT eyes was also increased, suggesting that this larger SC retained its fully differentiated phenotype (*p* = 0.0109). **E** Hepta-ANGPT1 treatment had no effect on the morphology of the limbal superficial capillary plexus suggesting the effect was specific to Schlemm’s canal. **F** Some *Angpt1*ΔNC eyes treated with Hepta-ANGPT1 exhibited a sprouting endothelial plexus in place of an organized, canal-like structure. **G** Intraocular pressure was measured at 8, 10, and 15 weeks in mice that received Hepta-ANGPT1 from birth to P14. Wildtype *n* = 12 (vehicle) and 13 (Hepta-ANGPT1), *Angpt1*ΔNC *n* = 10 (vehicle) and 14 (Hepta-ANGPT1) from four independent litters. **H** Following IOP measurement, average Schlemm’s canal area was measured in whole mount eyes by confocal microscopy and **I** BRN3A-positive retinal ganglion cells were quantified in retina flat mounts of the same animals. **J** To examine the acute effect of Hepta-ANGPT1 on IOP in adult WT eyes, 8-week-old C57Bl/6J mice received bilateral intravitreal injections of either 1 μg Hepta-ANGPT1 or PBS vehicle after 3 days of baseline IOP measurement. IOP was then measured daily for 5 days following injection. *n* = 12 (vehicle) and 13 (Hepta-ANGPT1). Scale bars represent 100 μm, quantified fields represent a total area of 262,277 μm^2^ (**B**, **D**, **H**) and 65,413 μm^2^ (**I**). AFU, background-subtracted arbitrary fluorescence units. Dashed lines in PROX1 panels indicate CD31 + SC area. *p < 0.05, **p < 0.01, ***p < 0.001 as determined by two-tailed Welch’s *t*-test (**B**, **D**) Df = 9 (WT, **B**) and 8 (*Angpt1*ΔNC, **D**) or two-way ANOVA followed by Bonferroni’s correction (**G**–**J**). Df_group_ = 3, *F*_group_ = 172.2 (**G**), Df_treatment_ = 1, *F*_treatment_ = 4.6 (**H**), Df_group_ = 3, F_group_ = 11.92 (**H**), Df_treatment_ = 1, *F*_treatment_ = 24.03 (**J**).

To test the efficacy of Hepta-ANGPT1 in enhancing SC development, we administered once-daily doses of 1 mg/kg body weight via intraperitoneal injection to wild-type mice beginning at birth and continuing until P14, when animals were euthanized and their eyes were prepared for whole-mount immunostaining. Confocal microscopy revealed that fusion protein treatment led to a dramatic increase in SC area (Fig. 8A, quantified in B. Control: Vehicle: 5.01 ± 0.1, Treated: 7.62 ± 5.6 10^4^ μm^2^/20x field, *p* = 0.0014). The enlarged SC observed in treated eyes expressed elevated levels of the terminally differentiated SC marker PROX1, indicating that the expanded canal had retained its molecular identity (Fig. 8A, quantified in B). In addition, vessels of the superficial vascular plexus were morphologically normal in treated eyes, suggesting that the effect was specific to SC (Fig. 8E).

Encouraged by the success of ANGPT1 mimetic treatment in WT eyes, we repeated the experiment using *Angpt1*ΔNC mice to investigate whether exogenous ANGPT1 mimetic could rescue the SC phenotype observed in these animals. As expected, eyes from vehicle-treated mice revealed a hypomorphic SC with reduced or absent PROX1 staining (Fig. 8C, quantified in D). However, treatment was sufficient to partially rescue SC development and we observed a dramatic increase in SC area in treated eyes, exhibiting a similar SC area to vehicle-treated WT eyes (*Angpt1*ΔNC: Vehicle: 2.93 ± 1.0, Treated: 4.93 ± 3.7 10^4^ μm^2^/20x field, *p* = 0.0008). Despite the increase in canal area, most eyes showed no significant increase in overall PROX1 expression compared to untreated *Angpt1*ΔNC eyes. Although localized PROX1 expression was present in some regions, these findings suggested that the enlarged endothelial structure induced by ANGPT1-mimetic in *Angpt1*ΔNC mice was not a fully mature SC. In addition, instead of forming a coherent canal, some *Angpt1*ΔNC eyes treated with Hepta-ANGPT1 exhibited a focally disorganized endothelial plexus resembling the sprouting morphology of early SC development (Fig. 8F). To determine if developmental Hepta-ANGPT1 treatment had a lasting effect on outflow pathway function in adult mice after cessation of treatment, an additional cohort of *Angpt1*ΔNC and littermate mice was generated and treated with Hepta-ANGPT1 or vehicle from P0-P14. IOP was then measured at 8, 10, and 15 weeks of age, revealing reduced IOP in Hepta-ANGPT1 treated *Angpt1*ΔNC mice when compared with vehicle-treated controls even 13 weeks after treatment was halted (Fig. 8G). No change in IOP was observed in Cre-negative mice. Consistent with these results, at 15 weeks of age *Angpt1*ΔNC animals that had been treated with Hepta-ANGPT1 retained the enlarged SC seen at P14 when compared to vehicle-treated animals of the same genotype (Fig. 8H, Supplementary Fig. 9, Hepta-ANGPT1: 4.70, vehicle: 3.52 10^4^ μm^2^/field, *P* < 0.01). No difference was observed in the size of SC in wild-type mice (Hepta-ANGPT1: 6.78, vehicle: 6.82 10^4^ μm^2^/20x field). Loss of BRN3A-positive RGCs was blunted in the peripheral retina of treated mutants (Fig. 8I, Supplementary Fig. 10, Hepta-ANGPT1: 201.6, vehicle: 153.7 BRN3A+ nuclei/field, *P* < 0.01), demonstrating the persistent functional effect of neonatal Hepta-ANGPT1 treatment.

While PCG is devastating to those affected, it represents a small fraction of worldwide glaucoma. To investigate the potential of Hepta-ANGPT1 as a therapy for POAG, and explore the effects of localized treatment, 1 μg of Hepta-ANGPT1 or PBS vehicle was delivered to the eyes of adult male C57Bl/6J mice by bilateral intravitreal injection. 24 h after injection, a marked reduction in IOP was observed in the Hepta-ANGPT1 treated group compared to either the vehicle-treated controls or to their pre-treatment baseline (Fig. 8J, Baseline: Vehicle group 13.98 ± 0.41 mmHg, Hepta-ANGPT1 13.57 ± 0.29 mmHg, 24 h post injection: Vehicle 13.67 ± 0.45 mmHg, Hepta-Angpt1 10.12 ± 0.56 mmHg, *p* < 0.001). IOP in the Hepta-ANGPT1 treated group remained significantly lower than the vehicle-treated controls for 60 h after injection before returning to baseline by the 72 h timepoint. No effect on SC, limbal capillary or superficial retinal vascular morphology was observed 7 days after Hepta-ANGPT1 injection (Supplementary Fig. 11).

### Genetic deletion of the phosphatase PTPRB activates TEK but does not restore SC

As an alternative to ANGPT1-mimetics, inhibition or genetic deletion of PTPRB (VE-PTP), which negatively regulates the TEK receptor, has been studied in animal models and is currently being tested in adult patients with glaucoma. To compare the efficacy of *Ptprb* deletion with Hepta-ANGPT1 treatment during SC formation, we generated an inducible *Ptprb* knockout mouse model using the endothelial-specific *Cdh5-*CreERT2^49^ to excise *Ptprb* from the endothelium of *Ptprb*^flox/flox^ mice^50^ at P0 (*Ptprb*ΔEC-P0 mice), analogous to our studies with Hepta-ANGPT1. As above, mice were dissected at P14, and eyes were prepared for analysis. Unlike control mice treated with Hepta-ANGPT1, no increase in SC size was observed in *Ptprb*ΔEC-P0 mice compared to Cre-negative littermates (Supplementary Fig. 12A, B). However, a marked increase in diameter of the superficial limbal vessels was observed (Supplementary Fig. 12C), which was not seen in Hepta-ANGPT1-treated mice (Fig. 8E). Next, to investigate the effect of *Ptprb* deletion in the context of *Angpt1* knockout, we generated an additional mouse line using the whole-body doxycycline-inducible *Rosa26-*rtTA-TetOCre system. Use of this additional Cre-driver system was necessitated by the divergent expression patterns of *Ptprb* and *Angpt1* in the endothelial and uveal tissues of the eye, respectively. Doxycycline was provided in the drinking water of pregnant dams at E16.5 to generate *Angpt1*;*Ptprb*ΔE16.5 mice. As expected, analysis at P14 revealed reduced SC area in mice lacking *Angpt1* (Supplementary Fig. 12D, E). However, as in *Ptprb*ΔEC-P0 mice, parallel deletion of *Ptprb* had no effect on SC area. In addition, and consistent with our single-cell RNA sequencing results (Fig. 6D), we observed lower expression of a *Ptprb*-NLS-LacZ transgene in SC compared with the adjacent blood capillaries (Supplementary Fig. 12F), suggesting a potential mechanism for the differential response of SC and blood vascular endothelium to *Ptprb* deletion.

## Discussion

Cells of the TM have well established roles in filtration of the aqueous humor and regulation of outflow resistance. However, they also have a critical signaling role, providing growth factors and other molecules required for the development and regulation of the SC endothelium. In return, SC derived signaling molecules regulate the TM, forming a reciprocal system analogous to the relationship between vascular endothelial cells and their supporting mural cells. Elsewhere in the body, disruptions in this relationship lead to defects in vascular development and play a central role in diseases ranging from diabetic retinopathy to kidney disease and cancer^51^. Several well-characterized EC-mural signaling pathways, including the angiopoietin, VEGF^4,5,52^, TGFB^53^ and BMP^54,55^ pathways have been linked to glaucoma or elevated IOP in humans and animal models. These findings suggest that signaling interactions between SC and nearby supporting cells are essential for IOP homeostasis. Here, we tested tissue specific deletion of *Angpt1* and *Svep1* in cells of neural crest origin to investigate the importance of uveal-SC crosstalk in SC development, function and IOP homeostasis.

The role of ANGPT1-TEK signaling in SC development^14,18^, and function in rodents and non-human primates have been well described^18,19^. In humans, variants in *ANGPT1* and *TEK* have been identified as a cause of PCG and associated with a higher risk of POAG, suggesting a similar role^13–17^. However, while ANGPT1 expression has been described in the TM and other cells adjacent to SC, previous studies have relied on whole-body deletion or blocking antibodies rather than tissue-specific deletion, leaving the cellular origin of ANGPT1 unverified. Using neural crest-specific *Wnt1*-cre, we have shown that deletion of *Angpt1* in the uveal tract recapitulates the glaucoma phenotype of whole-body knockouts, confirming the TM origin of SC-regulating ANGPT1. In comparison to our previous studies in whole body inducible *Angpt1* knockouts, neural-crest specific *Angpt1* knockout mice showed slightly higher IOP at 10 weeks of age^56^ (*Angpt1*ΔNC: Control: 11.5, mutant: 21.2, Δ9.7 mmHg; *Angpt1*-whole body: Control: 15.4, mutant: 20.9, Δ5.5 mmHg), possibly due to strain differences or improved excision of TM *Angpt1* in *Angpt1*ΔNC mice.

### The role of Svep1 in SC development

SVEP1, also known as Polydom, is a large ECM protein containing a von Willebrand Factor A domain, a pentraxin domain, 10 EGF-like domains and 34 complement control protein modules^57^. It has an evolutionarily conserved role in lymphatic development in zebrafish and mammals, with homozygous *Svep1* knockout embryos exhibiting edema with failure of lymphatic remodeling and valve development^20,21^. SVEP1 is expressed by lymphatic-associated mesenchymal cells, from where it is deposited in a fibrillar pattern to serve as a ligand for lymphatic endothelial cell-expressed integrin α9β1, encoded by the genes *Itga9* and *Itgb1*^20,35^. This mechanism appears to be conserved in the iridocorneal angle, where our data suggest that *Svep1* expressed by neural crest-derived cells of the TM is essential for the development of the integrin α9β1-expressing SC. While we anticipated SC and lymphatic vessel phenotypes, we were surprised to discover abnormal patterning in the limbal vascular network as to our knowledge blood vascular defects have not previously been reported in mice deficient in Svep1. However, defects in venous sprouting have been described in Svep1-deficient zebrafish^21^, and it is possible that a similar effect was present in the iridocorneal angle—although normal blood vascular morphology was observed in the skin of *Svep1*^null/null^ embryos (Supplementary Fig. 4).

In addition to its direct signaling role, in vitro studies have shown that SVEP1 can bind to ANGPT1 and ANGPT2, and reduced *Tek* mRNA expression was detected in lymphatic endothelial cells of *Svep1*-null mice^20,21^. This raised the possibility of interaction between the ANGPT-TEK and SVEP1 pathways, and offered a potential mechanism for the role of *SVEP1* variants both as late-onset glaucoma risk alleles and as a modifier of penetrance and severity in a multi-generation affected family with TEK-related PCG^22^.

Interestingly, while SVEP1 appears to have the highest affinity for integrin α9β1, other ligands have been identified and are also highly expressed in the TM including Tenascin-C, Emilin-1^58^, and Osteopontin^59^. None of these proteins have been linked to elevated IOP, although Tenascin-C expression is regulated by aqueous humor outflow^41^, and Osteopontin knockout mice exhibit RGC loss through an IOP-independent mechanism^60^.

### Cellular signaling in the SC and TM

The relationship between SC and the neural-crest derived TM is central to the regulation of aqueous humor outflow. However, the molecular identity of these cells and the signaling pathways used to communicate between them remain poorly understood. Recently, multiple groups have used the newly emerging technology of single-cell RNA sequencing to address this critical knowledge gap with an eye towards the discovery of new drug targets and glaucoma-relevant genes^39,61^. Here, we have expanded on these studies, using the hypomorphic SC in *Angpt1*ΔNC mice as an independent verification of SC endothelial cells in our single-cell transcriptome dataset. This strategy has allowed us to positively distinguish the *Lyve1*-negative SC and *Lyve1*, *Pdpn*-positive LECs, and to identify additional markers differentiating these cell populations, including *Pgf*, *Npnt,* and *Postn* (SC) as well as *Nts* and *Fgl2* (LEC). These results made clear that, while SC endothelial cells display aspects of the lymphatic phenotype, their overall transcriptome is more closely related to blood vascular endothelial cells. Identification of SC and TM cell populations has also allowed us to explore putative receptor-ligand pairs, which might be involved in IOP homeostasis and glaucoma, including SVEP1 and integrin α9β1, and will provide a resource for hypothesis generation in concert with available large-scale genetics studies which have identified IOP or glaucoma-associated loci.

### TEK activation as a therapeutic strategy

Although the majority of aqueous humor outflow occurs through the conventional route^62^, most current standard of care pharmacological glaucoma therapies target the unconventional outflow pathway or seek to reduce the production of aqueous humor. However, a greater understanding of SC molecular identity, development, and function has allowed researchers to develop approaches targeting SC and the conventional outflow directly, either through broadly active compounds such as Rho kinase inhibitors, actin depolymerizers, and other stiffness-altering drugs^63,64^ or endothelial-specific signaling pathways^5,18,65,66^. Of strategies targeting endothelial signaling, work exploiting the ANGPT-TEK pathway is among the most advanced with several approaches under investigation.

Here, we have shown that treatment of *Angpt1*ΔNC mice with intra-peritoneal Hepta-ANGPT1 from birth to P14 partially compensated for the loss of endogenous ANGPT1 and allowed the development of a functional SC. Despite the discontinuation of treatment at P14, adult Hepta-ANGPT1 treated mice had reduced IOP compared to vehicle-treated *Angpt1*ΔNC controls, increased SC size and blunted loss of RGCs, indicating lasting effect of this developmental rescue. In WT mice, developmental Hepta-ANGPT1 boosted SC development, leading to an enlarged SC with elevated PROX1 expression. Retention or increase in PROX1 expression in treated eyes was important confirmation that the enlarged canal retained its differentiated phenotype and was conducting outflow as PROX1 is an important marker of the differentiated SC phenotype and reduced aqueous humor outflow leads to decreased PROX1 expression^34^.

As systemic Hepta-ANGPT1 was unlikely to cross the blood aqueous barrier and reach SC in adult mice, we used intravitreal delivery to introduce the protein directly into the eye of adult animals. In rabbits, a recent pharmacokinetic study demonstrated high levels of Hepta-ANGPT1 in the aqueous humor for 3 days after intravitreal delivery^48^, and our data suggest that a similar time course occurs in mice. Providing further evidence for the potential of TEK activation as an IOP lowering strategy, IOP reduction has been reported after treatment with other TEK activators, including the ANGPT2-clustering antibody ABTAA^18^ and the PTPRB inhibitor AKB-9778/razuprotafib^66^. Twenty-four hours after treatment, we found that Hepta-ANGPT1 lowered IOP as much or more than these other approaches in adult mice with some important differences: ABTAA was reported to lower IOP only in *Angpt1*;*Angpt2* double knockout mice and not in WT animals^18^ and while AKB-9778/razuprotafib treatment led to an acute decrease of 5.66 mmHg 2 h after treatment, this effect was short lived and treated eyes were only 1.6 mmHg lower than vehicle-treated controls 24 h after dosing. In contrast, 24 h after injection, Hepta-ANGPT1-treated mice exhibited an IOP decrease of 3.45 mmHg compared to their pre-treatment baseline and 3.55 mmHg compared to vehicle-treated controls, an effect that persisted for 60 h after treatment.

Unlike treatment with VEGFC which also leads to canal remodeling^5^, Hepta-ANGPT1 treatment did not induce pathological angiogenesis of the canal or other limbal vessels either during development or after intravitreal injection of adult mice. Furthermore, Hepta-ANGPT1 did not induce the dramatic limbal capillary widening we observed in *Ptprb* knockout mice although both approaches lead to elevated TEK activation. However, as limbal capillary morphology was not reported in prior studies, it is unclear if this widening also occurred after AKB-9778/razuprotafib treatment or if it is specifc to *Ptprb* knockout mice.

While Hepta-ANGPT1 treatment had a dramatic effect on SC development in both WT and *Angpt1*ΔNC eyes, the failure of *Ptprb* deletion to rescue the phenotype of the *Angpt1* knockout PCG model suggests that ligand-dependent TEK activation is required for normal SC formation. While *Ptprb* deletion results in potent TEK activation^50,67^, interaction with an angiopoietin ligand is a critical aspect of TEK receptor trafficking. Following stimulation with oligomerized ANGPT1 or Hepta-ANGPT1, TEK is rapidly translocated to the cell-cell junction^48,67^. This behavior plays a key role in the regulation of downstream signaling events, promoting AKT/ENOS signaling while suppressing the Dok-R and ERK pathways^68,69^. PTPRB inhibition leads to increased activation but does not trigger this translocation, potentially resulting in differential downstream signaling events^67^. In addition, these differences may be exaggerated by the comparatively lower *Ptprb* expression observed in SC compared with the limbal blood vasculature.

Together, our results demonstrate that ANGPT1 and SVEP1 are examples of a large group of TM-derived molecules, which are essential regulators of SC development and function. Emphasizing the potential of these signaling molecules as novel glaucoma therapies, we found that an ANGPT1-mimetic could specifically induce developmental SC remodeling and growth in wild-type and *Angpt1*ΔNC eyes and lower IOP in healthy adult eyes without pathological angiogenesis. In addition, several other signaling molecules identified in our single-cell transcriptomics dataset have been implicated in glaucoma and offer exciting opportunities for future studies and drug development directly targeting the conventional outflow pathway.

## Methods

### Study approvals

Animal experiments were approved by the Animal Care and Use Committee at Northwestern University (Evanston IL, USA) and comply with ARVO guidelines for care and use of vertebrate research subjects in Ophthalmology research.

### Animal generation and husbandry

Throughout the study, mice were housed at the Center for Comparative Medicine of Northwestern University (Chicago, IL, USA). Animals were maintained on a standard 12 h lighting cycle in a vivarium maintained at 21–23 °C, relative humidity of 30–70% and received unrestricted access to standard mouse chow (Teklad #7912, Envigo, Indianapolis, IN) and water.

To generate neural crest-specific *Angpt1* knockout (*Angpt1*ΔNC) mice, previously described *Angpt1*-floxed mice (Angpt1^tm1.1Seq^)^31^ were crossed with a previously published *Wnt1*-Cre expressing mouse line (B6.Cg-H2az2^Tg(Wnt1-cre)11Rth Tg^(Wnt1-GAL4)11Rth/J)^26^. Throughout the study, *Angpt1*^flox/flox^ mice were crossed with *Angpt1*^flox/flox^;Wnt1-Cre^+^ animals to generate litters of *Angpt1*^flox/flox^;Wnt1-Cre^+^ knockouts and *Angpt1*^flox/flox^ controls. In addition to *Angpt1*^flox/flox^ animals, Wnt1-Cre mice were also crossed to Gt(ROSA)26Sor^tm4(ACTB-tdTomato,-EGFP)Luo^/J (Rosa26^mTmG^, JAX #007576) mice to examine the localization of neural crest-derived cells in the anterior chamber^27^. *Angpt1*-GFP (Angpt1^tm1.1Sjm^) mice were a generous gift of Dr. Sean Morrison (UT Southwestern Medical Center) and were genotyped by PCR as previously described^24^. *Ptprb*^NLS-LacZ/WT^ mice have been previously described^70,71^ and were a gift of Dr. Dietmar Vestweber (Max Planck Institute, Münster, Germany). Doxycycline-inducible *Angpt1*;*Ptprb*ΔE16 mice were generated by breeding with *Rosa26*^rtTA^ (Gt(ROSA)26Sortm1(rtTA,EGFP)Nagy) and TetOCre-expressing mouse lines as previously described^72^. Gene deletion at E16.5 was achieved by addition of doxycycline hyclate (0.5%; Sigma-Aldrich) to the drinking water of pregnant dams. Doxycycline treatment was continued until P14. Endothelial-specific *Ptprb* deletion in *Ptprb*ΔEC-P0 mice was achieved by daily intraperitoneal administration of 200 μg tamoxifen (Sigma-Aldrich) in corn oil to *Ptprb*^flox/flox^; *Cdh5*CreERT2 (Tg(Cdh5-cre/ERT2)^1R*ha*^)^49^ from P0-P3. All mice were maintained on a mixed genetic background free of the RD1 and RD8 mutations and animals of both sexes were included in comparisons. Knockout mice were genotyped as previously described^26,31^. All primers used for PCR genotyping are provided in Supplementary Table 2.

### IOP measurements

IOP measurements were performed in awake mice between 9 and 11 AM using a Tonolab rebound tonometer as previously described^73,74^. Cohorts of mutant mice with littermate controls were measured at each reported timepoint. IOP values from left and right eyes were averaged to obtain values reported in the manuscript.

### Hepta-ANGPT1 treatment

Hepta-ANGPT1 was generated and purified as previously described^48^. Littermate female mice were plugged by the same male and beginning at birth full litters received once-daily intraperitoneal injections of either 1 mg/kg body weight Hepta-ANGPT1 or sterile PBS vehicle until P14. At P14 animals were euthanized and eyes processed for whole-mount imaging of SC as described below or aged to 8 weeks for IOP measurement.

For adult studies, male C57Bl/6J mice were purchased from The Jackson Lab (Bar Harbor Maine, USA) and allowed to acclimate to our colony. At 8 weeks of age mice were randomized into groups and 3 days of baseline IOP measurements were taken, followed by a single, bilateral, intravitreal injection of 1 μg Hepta-ANGPT1 or PBS vehicle under ketamine/xylasine anesthesia supplemented with 0.5% proparacaine hydrochloride eyedrops (Alcon Laboratories, Ft. Worth TX, USA). After injection eyes were treated with ophthalmic triple antibiotic ointment and mice were allowed to recover. Beginning 24 h after injection, once-daily IOP measurements were resumed for 5 days. Mice were then euthanized for histological analysis.

### Generation of Svep1 knockout mice

The *Svep1*^tm1a(EUCOMM)Hmgu^ line was generated by the Knockout Mouse Project (KOMP) Repository of the University of California (Davis, CA, https://www.komp.org/ProductSheet.php?cloneID=861546). In the targeted allele, a knockout-first beta-galactosidase reporter cassette was introduced between exons 7 and 8 of the *Svep1* gene, with an additional loxP site engineered downstream of exon 8. Frozen sperm was purchased from EUCOMM and cryo recovery was performed by the Northwestern University Transgenic and Targeted Mutagenesis Laboratory at the Feinberg School of Medicine. After confirming germline transmission of the *Svep1*^tm1a(EUCOMM)Hmgu^ allele, mice were bred to B6;SJL-Tg(ACTFLPe)9205Dym/J (Jax stock No: 003800) to remove the neomycin cassette. After neomycin cassette removal, loxP sites flank exon 8 of the *Svep1* locus. Excision of exon 8 results in a frameshift mutation, resulting in a conditional allele. *Svep1*-floxed mice were then bred to EIIa-Cre (Tg(EIIa-cre)C5379Lmgd)-expressing mice to generate *Svep1*^null^ and to Wnt1-Cre expressing mice to generate neural crest specific knockouts (*Svep1*ΔNC).

### Quantitative rtPCR

*Angpt1* expression was analyzed in *Angpt1*ΔNC and control littermate eyes at 12 weeks of age. After euthanasia, eyes were enucleated and microdissected into fractions consisting of posterior eyecup (containing sclera, choroid and RPE), limbus/iridocorneal angle (TM, SC, ciliary body), and retina/vitreous. Iris tissue was generally excluded from the limbus/iridocorneal angle fraction, although in some samples peripheral iris may have been retained in the interest of preserving the TM/ciliary body. Tissues from left and right eyes were pooled before RNA purification. Lung tissue was also collected as a control. Total RNA was purified using Trizol reagent (Life Technologies, Carlsbad CA) according to the manufacturer’s directions. 500 ng total RNA was used for reverse transcription (iScript kit, Bio-Rad, Hercules CA). Real-time PCR was performed using an ABI 7500 thermo cycler and SYBR green master mix (iTaq, Bio-Rad, Hercules CA). All primers used are provided in Supplementary Table 2.

### Quantification of RGCs

RGCs were quantified as previously described^56^. Briefly, enucleated eyes were immersion fixed (2% formaldehyde in phosphate-buffered saline, pH 7.5) before retinas were dissected and blocked (5% donkey serum, 2.5% BSA, 0.5% Triton X100 in Tris buffered saline pH 7.5, overnight at 4°). Retinas were then incubated overnight (4°) in primary antibodies diluted in additional blocking buffer, washed, and subjected to a final overnight incubation in appropriate alexafluor-labeled secondary antibodies (ThermoFisher Scientific, Waltham, MA, USA. Donkey anti-mouse 488, #A21202; donkey anti-goat 647, #A21447). Images were captured using a Nikon A1R confocal microscope and 20x objective with a numeric aperture of 0.75 and a pinhole size of 44.70 μm. Values reported in the manuscript represent averages obtained from 3 to 4 imaging fields. Primary antibodies: Mouse anti-BRN3A (Millipore; MAB1585; 1:400), goat anti-BRN3B (Santa Cruz Biotechnology; sc-6026; 1:400) and rabbit anti-TUBB3 (Covance; MRB-435P; 1:1000). Retinal diameter was measured from flat mount images using ImageJ Fiji software.

### Analysis of retinal vasculature

As described above, retina flat mounts were prepared from enucleated, formaldehyde-fixed eyes and blocked overnight. Retinas were then incubated with rat anti mouse CD31 antibody (BD; #553370; 1:100), washed and labeled with alexafluor-conjugated secondary antibodies (ThermoFisher, donkey anti-rat 594, #A21209). For quantification of retinal angiogenesis, full images of P5 retinas were collected using a Nikon A1R confocal microscope by stitching fields collected using a ×10 objective with numerical aperture of 0.3 and a pinhole radius of 29.37 μm. ImageJ Fiji software was then used to measure distance from the optic nerve at both arterial and venous fronts. Datapoints reported in the manuscript are averages of 3–4 measurements taken from a single eye.

### Whole-mount imaging of SC

SC imaging was performed according to our previously published protocol^75^. After fixation as described above, limbal whole mounts were prepared and incubated overnight with rat anti-CD31 (BD; #553370; 1:100) and goat anti PROX1 (R&D systems AF2727; 1:250) primary antibodies at 4°. Samples were then washed, incubated with secondary antibody as above and mounted for imaging. Images were captured using a Nikon A1R confocal microscope equipped with a ×20 objective with a numeric aperture of 0.75 and a pinhole size of 44.70 μm. 10–15-image Z stacks were obtained with a step size of 1.67 μm and maximum intensity projections generated using Fiji software are shown in the manuscript. For quantification, three 15-image stacks were collected at intervals around the circumference of the eye, and total fluorescence projections obtained using the “Sum Slices” function in ImageJ. Quantification of background-subtracted PROX1 expression and SC area were obtained from these images and averaged to obtain the values reported.

### Analysis of skin and mesenteric lymphatic vessels

Skin and lymphatic vessels were stained as above, using rat anti-mouse CD31 antibody (BD; #553370; 1:100) and goat anti-VEGFR3 (R&D systems #AF349; 1:100) or goat anti-PROX1 antibodies (R&D systems AF2727; 1:250). Samples were then imaged using a Nikon A1R confocal microscope as above.

### Immunohistochemistry of human iridocorneal angle

Formalin-fixed human eye globes were obtained from the Eversight Foundation eye bank. Anterior chambers were isolated and 5 μm paraffin sections were prepared using standard methods. Sections were subjected to antigen retrieval (10 mM Tris, 1 mM EDTA, 0.05% Tween-20, pH 9, autoclaved 121 °C 30-min liquid cycle) before blocking (5% donkey serum, 2.5% BSA, 0.5% Triton X-100 in TBS, pH 7.5, 1 h at room temperature) and overnight incubation using primary and secondary antibodies. Antibodies used: Goat anti-mouse CD31 (R&D systems; #AF3628; 1:100), rabbit anti-human SVEP1 (Aviva; #ARP58239; 1:100).

### Single-cell RNA sequencing: sample preparation

To avoid melanin aggregation during tissue dissociation and sample preparation, *Angpt1*ΔNC mice were crossed for two generations onto WT ICR mice to obtain litters of albino control and mutant animals. Animals were then aged to 6 weeks and groups of 2–3 *Angpt1*ΔNC and control mice were euthanized for pooled tissue collection. Male and female animals were pooled for analysis, resulting in two pooled control samples and a single pooled *Angpt1*ΔNC sample. 4–6 enucleated eyes were immediately dissected in ice-cold HBSS (Gibco-Life Technologies, Carlsbad CA) to obtain the isolated limbal-iridocorneal angle region. First, eyes were bisected on the sagittal plane before the lens and retina were removed. The sclera was then removed by cutting immediately posterior to the ciliary body. Finally, the cornea was separated by cutting just anterior to the limbus, avoiding the TM. The majority of the iris was then removed, taking care to leave the ciliary body and TM intact. Limbal regions were then transferred to ice-cold DMEM containing 10% FBS and minced using fine scissors. After mincing, 1 mg/ml Collagenase A (Worthington, Lakewood NJ) and 10 μM Y27362 were added and tissues were transferred to a tissue culture incubator for 2 h at 37°/5% CO_2_. After incubation, tissues were washed 1× in PBS before incubation in 0.25% Trypsin solution (Gibco) containing 100 units/ml DNAse 1 (Roche, Basel, Switzerland) 10 μM Y27362 for 25 min at 37° with shaking and trituration. Dissociated cells were then washed 2× in DMEM and passed through a 40 μm cell strainer before resuspension in ice-cold HBSS containing 1% BSA at a concentration of 1000 cells/μl. Cells were then filtered a second time and immediately used for library preparation by the NuSeq core at the Northwestern University Feinberg School of Medicine using the 10x Genomics Chromium platform. Libraries were then sequenced using a HiSeq4000 instrument at a depth of 50,000 reads per cell.

### Single-cell RNA sequencing: Bioinformatics

After sequencing, reads were aligned using the 10x Genomics CellRanger pipeline and analyzed using the Seurat package (version 3.1.3) for R.

Data filtered to exclude genes detected in <2 cells per sample and cells expressing <200 unique genes, <1000 unique molecular identifiers and those where >10% of total reads mapped to mitochondrial genes. After filtering 2524 control and 1360 *Angpt1*ΔNC cells, our dataset consisted of 21,356 control and 7254 *Angpt1*ΔNC cells. Doublet detection and removal was then performed using the scDblFinder package in R^76^. After doublet removal, 19,236 control and 6772 *Angpt1*ΔNC predicted singlets were used for all subsequent analysis steps. Next, samples were integrated using an anchor-based CCA pipeline in Seurat and the 5000 most highly variable genes in the dataset^37^. Genes which were detected in only a single sample, genes encoding sex-specific transcripts *Xist*, *Ddx3y*, *Eif2s3y*, *Erdr1*, *Gm29650*, *Kdm5d*, *Uty*, and *Angpt1* were excluded from the anchor set and all downstream analysis. PCA was performed on the integrated dataset in 20 dimensions, and UMAP was used to visualize PCA-reduced data. Clustering was then performed in Seurat using a Louvain algorithm at a resolution of 0.5, resulting in 25 unique clusters.

To identify SC endothelial cells, cluster 6 (representing *Cdh5*+ putative endothelial cells) was isolated and the maxLik package^77^ was used to estimate the dimensionality of the subsetted data before reclustering in a new set of 20 PCs. Reexamining cluster 1, which expressed a number of lymphatic and SC markers including *Prox1*, *Ccl21a*, *Flt4*, and *Plvap*, with its own set of 18 PCs revealed two distinct populations which we postulated comprised SC and lymphatic endothelial cells based on their expression of marker genes. Identification of TM cell populations was conducted in the same manner as SC cells (above). Clusters 11, 12, 14, and 16 were isolated from the full dataset and their dimensionality was estimated using maxLik. 18 PCs were then selected and used to recluster the subsetted data.

### Statistical analysis

Analysis of physiological and histological data was performed using Prism 5 software (Graphpad Software, San Diego, CA, USA), or R version 3.6.1. Throughout the text, reported *P* values were obtained using a two-tailed Student’s *t*-test, two-tailed Welch’s *t*-test, one-way ANOVA followed by Tukey-Kramer test, or two-way ANOVA followed by use of Bonferroni’s method for multiple comparisons as appropriate. Tests used for specific data are noted in figure legends. *P* values of <0.05 were considered significant and are indicated using the following notation: *P < 0.05, **P < 0.01, ***P < 0.001. To compare the proportion of putative SC endothelial cells in control and *Angpt1*ΔNC samples, a bootstrap approach was used to repeatedly sample 209 endothelial cells (the number of total endothelial cells present in the *Angpt1*Δ NC sample after quality control filtering) from the control sample, with replacement. 10,000 samples were collected, and a violin plot showing percentage of SC endothelial cells in the simulated result is shown in Fig. 6E.

### Reporting summary

Further information on research design is available in the Nature Research Reporting Summary linked to this article.

## Supplementary information

Supplementary Information

Supplementary Dataset 1

Supplementary Dataset 2

Description of Additional Supplementary Files

Reporting Summary

## Data Availability

Single-cell sequencing data associated with Figs. 5–7, Table 1 and Supplemental Datasets 1 and 2 is available on the NCBI Gene Expression Omnibus (GEO, accession number GSE168200. Source data for all figures are provided with this paper.

## Code availability

Code used for data analysis in R is accessible via GitHub (https://github.com/benrthomson/Angpt1.Angle.SingleCell)^78^.
