# Supplementary material for: Cellular crosstalk regulates the aqueous humor outflow pathway and provides new targets for glaucoma therapies
Source: Nat Commun. 2021 Oct 18;12:6072. doi: 10.1038/s41467-021-26346-0 (PMC8523664; doi:10.1038/s41467-021-26346-0)
Supplement: Supplementary file 1 — Supplementary Information [file 41467_2021_26346_MOESM1_ESM.pdf]

Supplemental data for:

Cellular crosstalk regulates the aqueous humor outflow pathway and provides new targets for glaucoma therapies

Benjamin R. Thomson, Pan Liu, Tuncer Onay, Jing Du, Stuart W. Tompson, Sol Misener, Raj R. Purohit,Terri L. Young, Jing Jin and Susan E. Quaggin

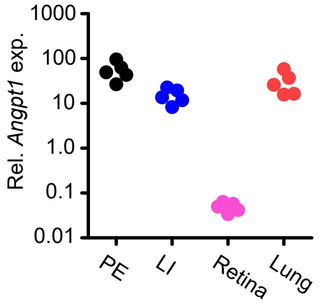

Supplemental figure 1. **Ocular expression of *Angpt1*.** When quantified using qRT-PCR and normalized to expression of *Gapdh,* ocular expression of *Angpt1* mRNA in wild-type adult mice was highest in tissues of the posterior eyecup (PE, choroid, sclera, retinal pigment epithelium) and limbus/iridocorneal angle (LI). In contrast, very low expression was detected in the retina. Lung tissue included as a comparative tissue with high *Angpt1* expression. n = 5 animals.

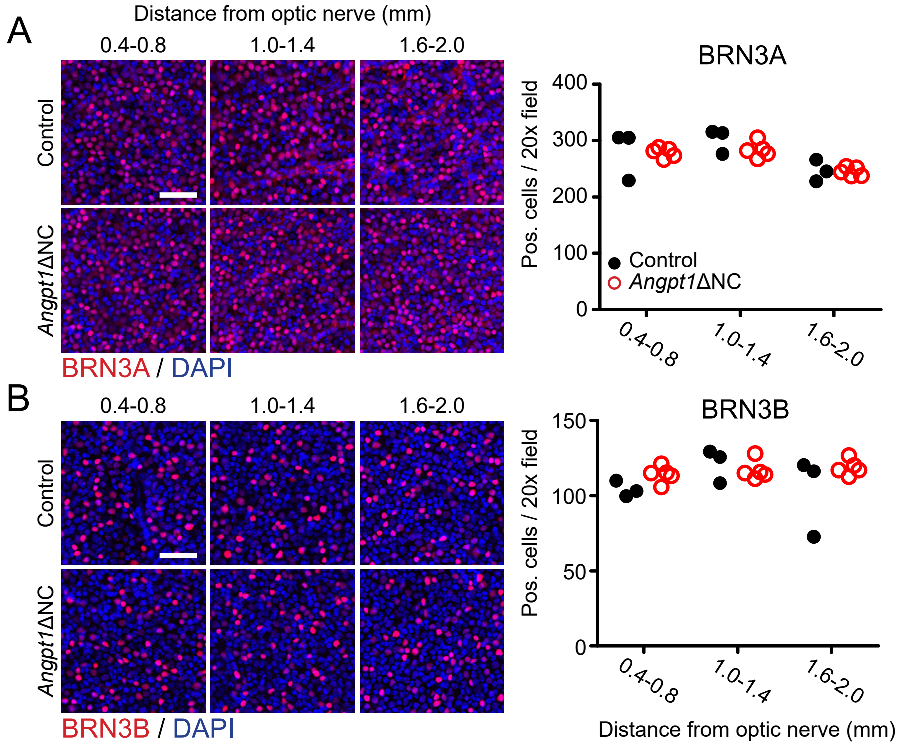

Supplemental Figure 2. *Angpt1*ΔNC mice exhibit normal retinal ganglion cell numbers at postnatal day 5 (P5). Quantification of (**A**) BRN3A and (**B**) BRN3B-positive RGCs at P5 revealed no difference in the number of RGCs between mutant mice and control littermates, confirming that reduction in RGCs observed in adult *Angpt1*ΔNC mice is the result of degeneration and not a developmental defect. Each datapoint represents average RGC number from a single animal. Lack of significance was assessed by 2-way ANOVA. n = 3 (control) and 5 (*Angpt1*ΔNC), $Df_{genotype} = 1$, $F_{genotype} = 0.7113$ (BRN3A), 2.39 (BRN3B). Scale bars represent 50 µm. 20x fields used for counting comprise an area of 65,025 µm^2.

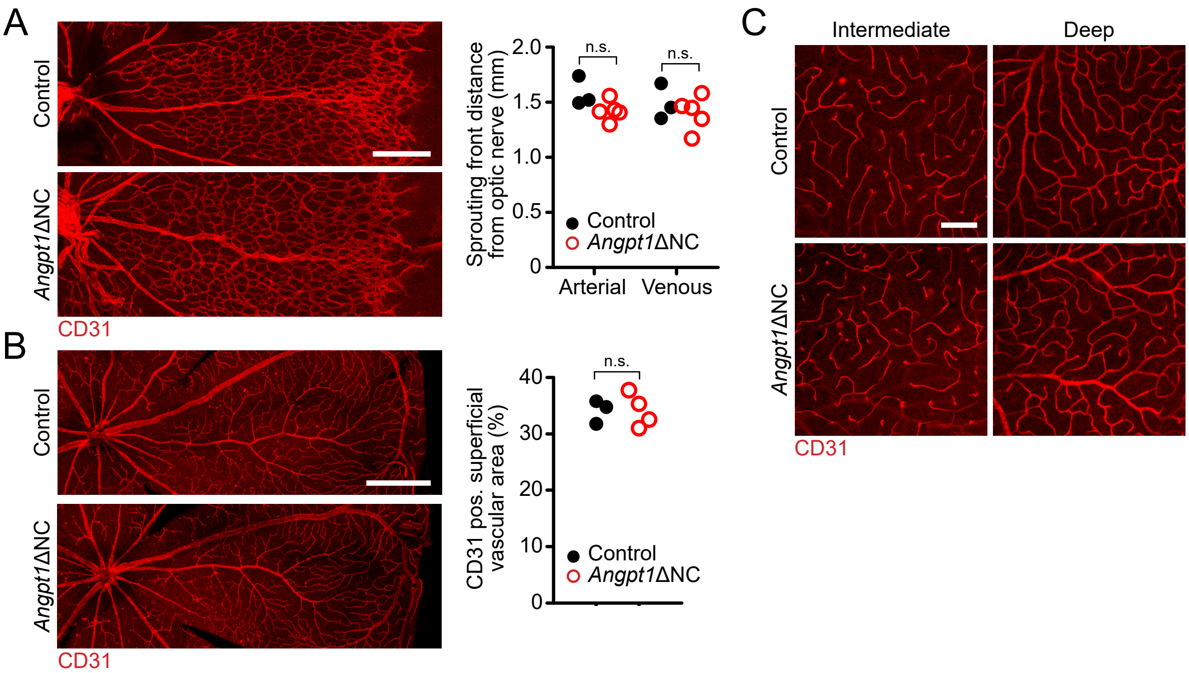

Supplemental Figure 3. Vascular patterning was normal in the retinas of *Angpt1*ΔNC mice. (**A**) CD31-stained retinal flat mounts showed no difference in angiogenic sprouting at either venous or arterial sprouting fronts between *Angpt1*ΔNC mice and control littermates when imaged at P5 using confocal microscopy. n = 3 (control) and 5 (*Angpt1*ΔNC). (**B**) At 15 weeks of age, vascular patterning and area was normal in the mature superficial vascular layer of Angpt1ΔNC retinas. n = 3 (control) and 4 (*Angpt1*ΔNC). (**C**) At 15 weeks of age, normal patterning was observed in the intermediate and deep vascular layers of all retinas quantified in (B). n.s. $p > 0.05$ as determined by 2-way ANOVA followed by Bonferroni's correction (A, $p_{genotype} = 0.1$, $Df_{genotype} = 1$, $F_{genotype} = 3.28$) or 2-tailed Student's t-test (B, $p = 0.97$, $Df = 5$). n = 3 (control) and 4-5 (Angpt1ΔNC). Scale bars represent 250 μm (A), 500 μm (B) and 100 μm (C).Shown are images and statistics from representative litters.

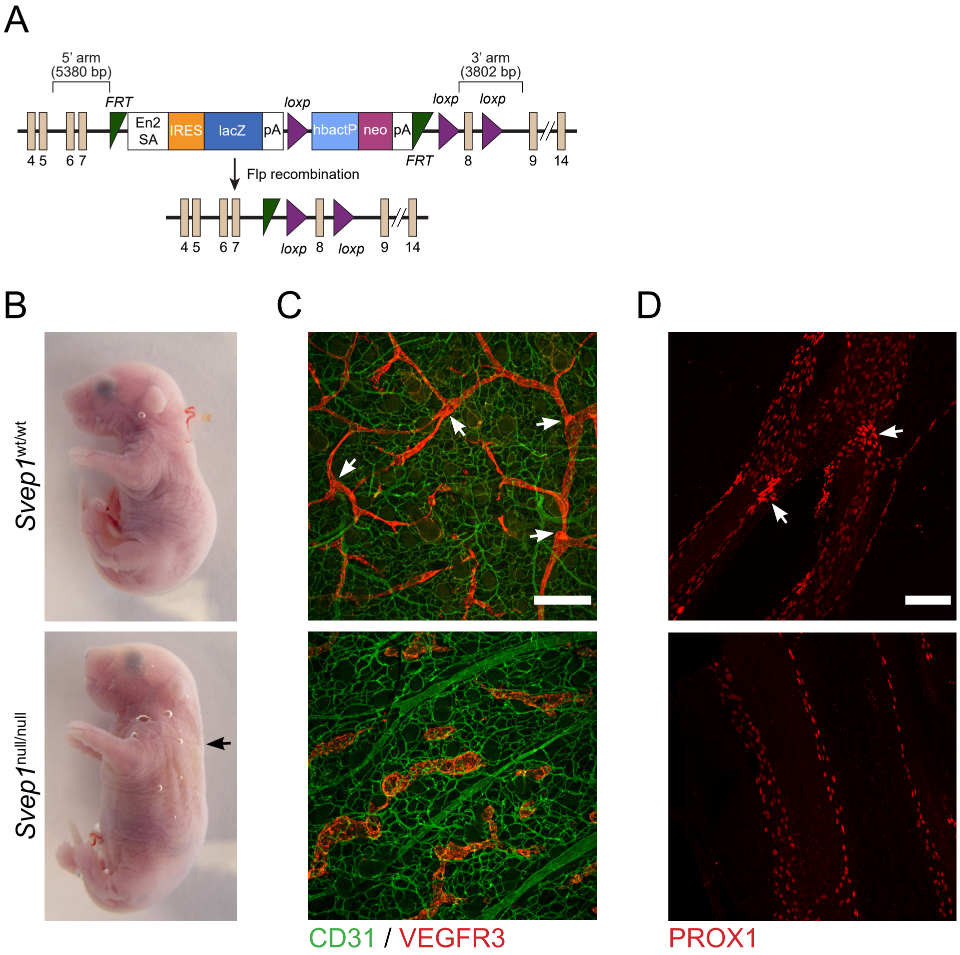

Supplemental Figure 4. (**A**) Cartoon illustrating the targeting strategy used to generate Svep1 floxed mice. Following Flp-mediated recombination to remove the neomycin selection cassette used in cloning, loxp sites flank exon 8 of the *Svep1* locus, which can then be excised by cre-mediated recombination. Deletion of exon 8 results in a frameshift and premature truncation of the protein. (**B**) As previously reported, *Svep1*$^{null/null}$ embryos were found alive at e18.5, but marked subcutanious edema was observed (black arrow). (**C**) While wildtype (WT) littermates exhibited organized lymphatic vasculature with regular valves (white arrows), whole mount staining of the dorsal skin revealed abnormal, dilated, lymphatic vessels lacking valves in mutant embroys. However, CD31-positive blood capillary patterning appeared normal. (**D**) Likewise, lymphatic valves were absent from the mesentery of *Svep1*$^{null/null}$ embryos. Scale bars represent 250 µm (C) and 100 µm (D). Representative embryos of each genotype from a litter of 12 pups are shown.

1: After removal of the lens and retina, limbal regions (red arrows) containing iridocorneal angle, ciliary body, limbus and peripheral iris, choroid, cornea and sclera were isolated from whole mouse eyes

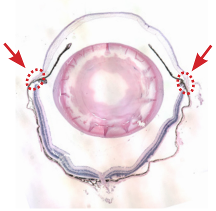

2: Pooled tissues were then dissociated using a combined Collagenase A / Trypsin protocol, filtered through a 40 µm cell strainer and immediately used for library preparation on the 10x Genomics Chromium platform

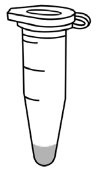

3: Libraries were sequenced at a depth of 50,000 reads per cell on a HiSeq 4000 instrument and aligned using CellRanger. Cellranger output was then analyzed using Seurat 3.1.3.

4: Quality control filtering was performed in seurat to remove droplets expressing <200 unique genes, <1000 UMIs or >10% mitochondrial transcripts

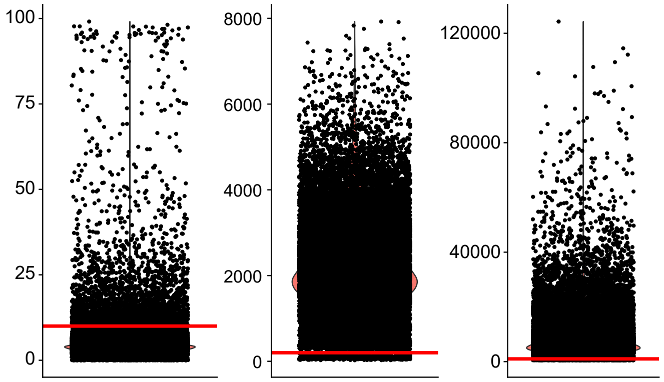

% Mitochondrial reads Cutoff: <10%

Unique transcripts Cutoff: >200

UMI Cutoff: >1000

	Cells		
	**Sequenced**	**Filtered**	**Analyzed**
Control	23,880	2,524	21,356
*Angpt1*ΔNC	8,614	1,360	7,254

5: scDblFinder was used to identify doublets in each sample. These putative doublets were removed and the predicted singlets were used for downstream clustering analysis in Seurat.

		scDblFinder prediction	
	**Total cells**	**Singlets**	**Doublets**
Control	21,356	19,236	2,120
*Angpt1*ΔNC	7,254	6,772	482

Supplemental Figure 5. Sample preparation and quality control strategy used for single cell RNA sequencing experiments.

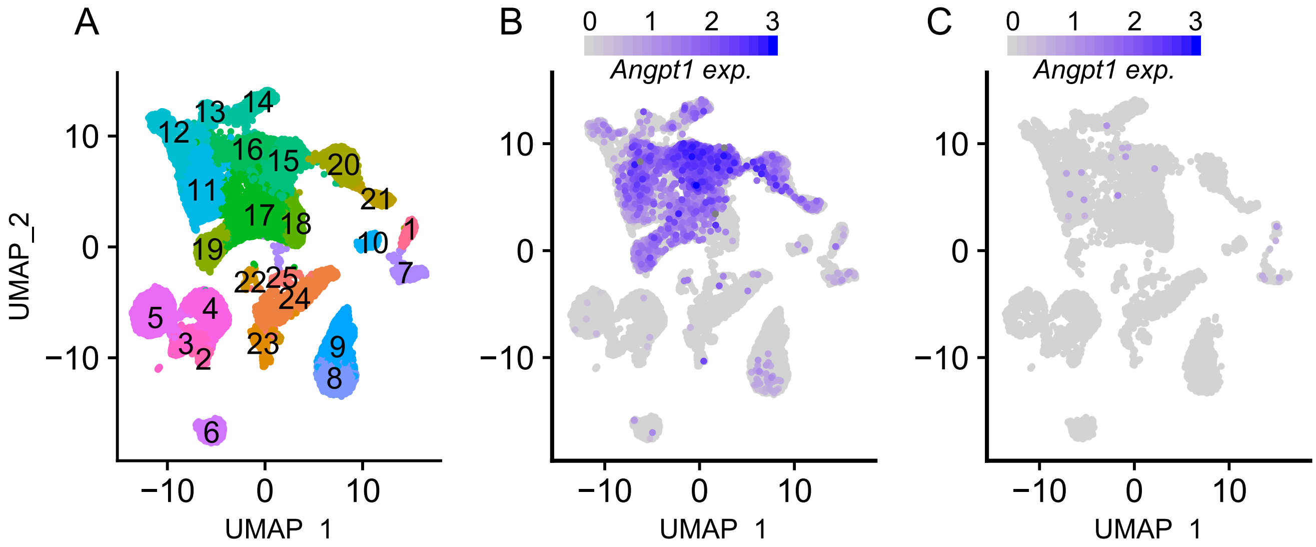

Supplemental Figure 6. The majority of *Angpt1*-expressing cells detected by single cell RNA sequencing of *Angpt1*ΔNC mice originate in clusters which natively express *Angpt1*. (**A**) UMAP projection illustrating labeled clusters reproduced from Figure 6 A. (**B**) *Angpt1* expression in WT samples indicates robust expression in uveal stromal clusters. (**C**) Nearly complete loss of *Angpt1* expression in *Angpt1*ΔNC samples indicates that all *Angpt1*-expressing cells of the limbal-iridocorneal angle region are derived from the neural crest. Residual *Angpt1*-expressing cells were observed only within NC-derived, *Angpt1*-expressing clusters, indicating that these cells represent incomplete cre-mediated excision and not the presence of *Angpt1* outside the neural crest lineage.

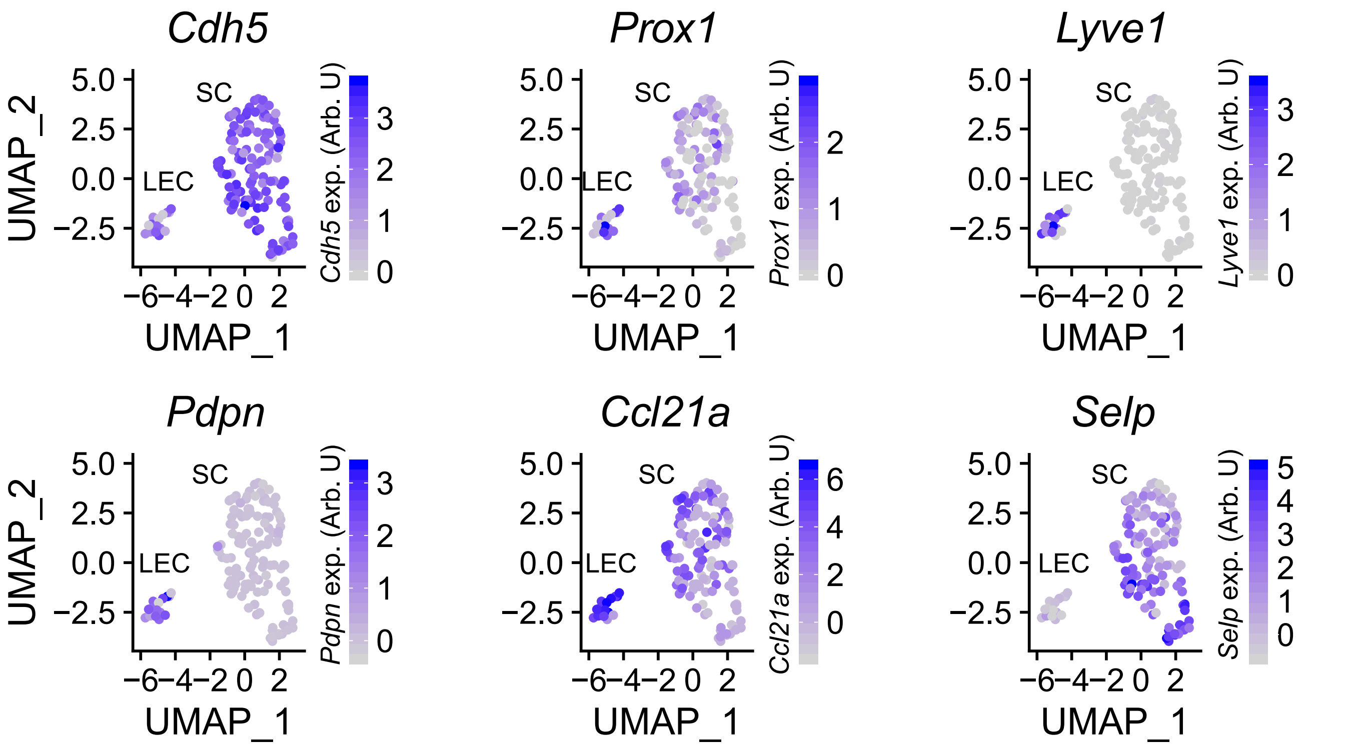

Supplemental Figure 7. Expression of Schlemm's canal (SC, *Cdh5, Prox1, Ccl21a, Selp*) and lymphatic endothelial cell (LEC, *Cdh5, Prox1, Lyve1, Pdpn, Ccl21a*) marker gene transcripts in putative SC and LEC endothelial cell clusters identified by single cell RNA sequencing.

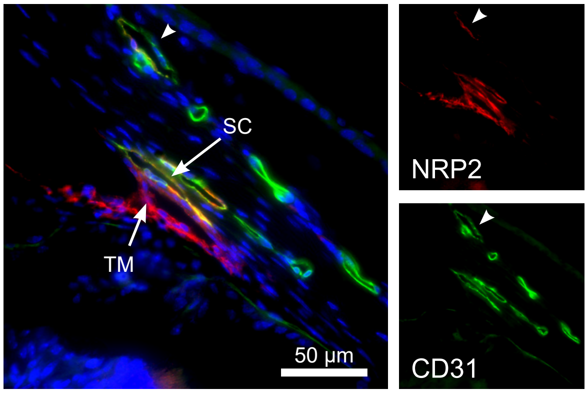

Supplemental Figure 8. Immunostaining revealed robust Neuropilin-2 (NRP2) expression in the mouse TM as well as SC endothelium. Note the NRP2-positive limbal lymphatic (white arrowhead). Representative section from a group of 3 wild-type eyes shown.

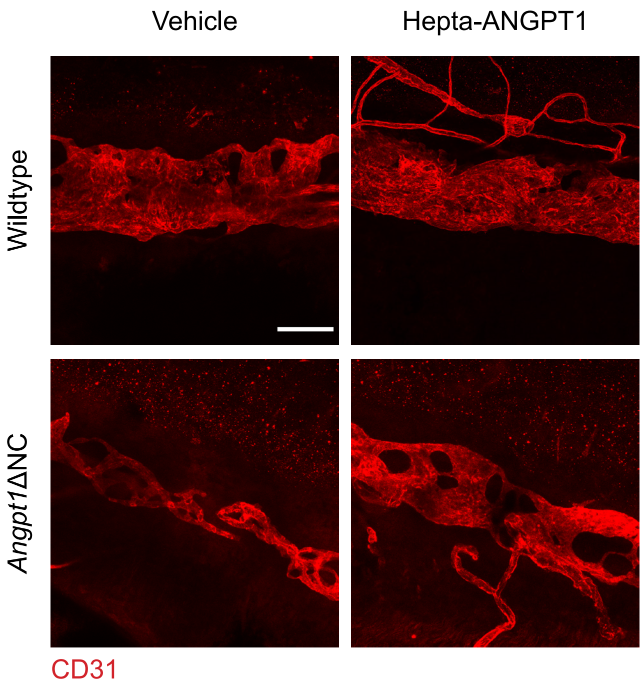

Supplemental Figure 9. Compared to vehicle-treated controls, immunostaining of Schlemm's canal at 15 weeks of age revealed increased canal size in *Angpt1*ΔNC mice that had been treated with daily Hepta-ANGPT1 from birth to P14. Representative images from the quantified data shown as Figure 8 H. Wildtype n = 12 (vehicle) and 13 (Hepta-ANGPT1), Angpt1ΔNC n = 10 (vehicle) and 14 (Hepta-ANGPT1) from 4 independent litters. Scale bar represents 100 μm.

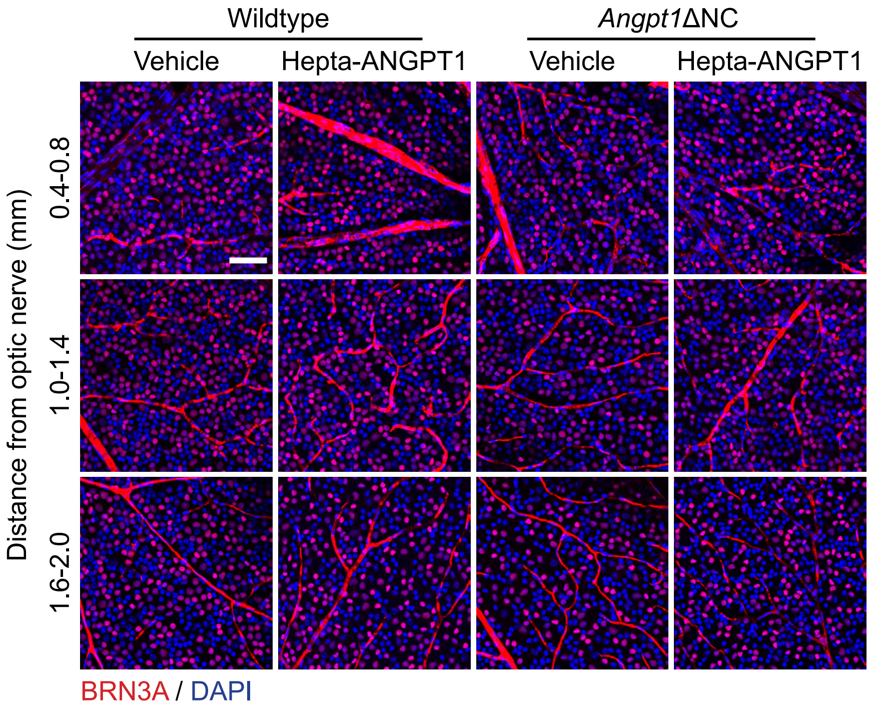

Supplemental Figure 10. Compared to vehicle-treated controls, immunostaining of retinal flat mounts at 15 weeks of age revealed blunted loss of BRN3A-positive retinal ganglion cells (RGCs) in *Angpt1*ΔNC mice that had been treated with daily Hepta-ANGPT1 from birth to P14. Representative images from the quantified data shown as Figure 8 I. Scale bar represents 50 μm. Wildtype n = 12 (vehicle) and 13 (Hepta-ANGPT1), Angpt1ΔNC n = 10 (vehicle) and 14 (Hepta-ANGPT1) from 4 independent litters.

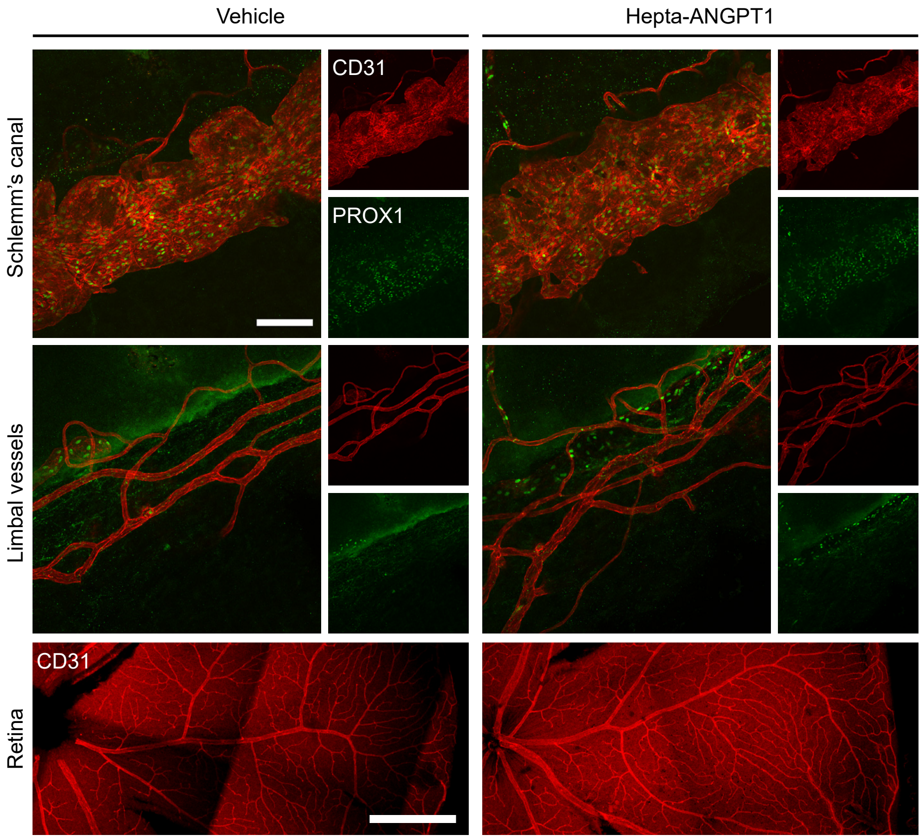

Supplemental Figure 11. No change in Schlemm's canal, limbal capillary or superficial retinal vascular morphology was observed 1 week after a single intravitreal injection of 1 µg Hepta-ANGPT1. Scale bar indicates 100 µm (SC and limbus) and 500 µm (retina). Representative images from histology of the mice described in figure 8 J, n = 12 (vehicle) and 13 (Hepta-ANGPT1).

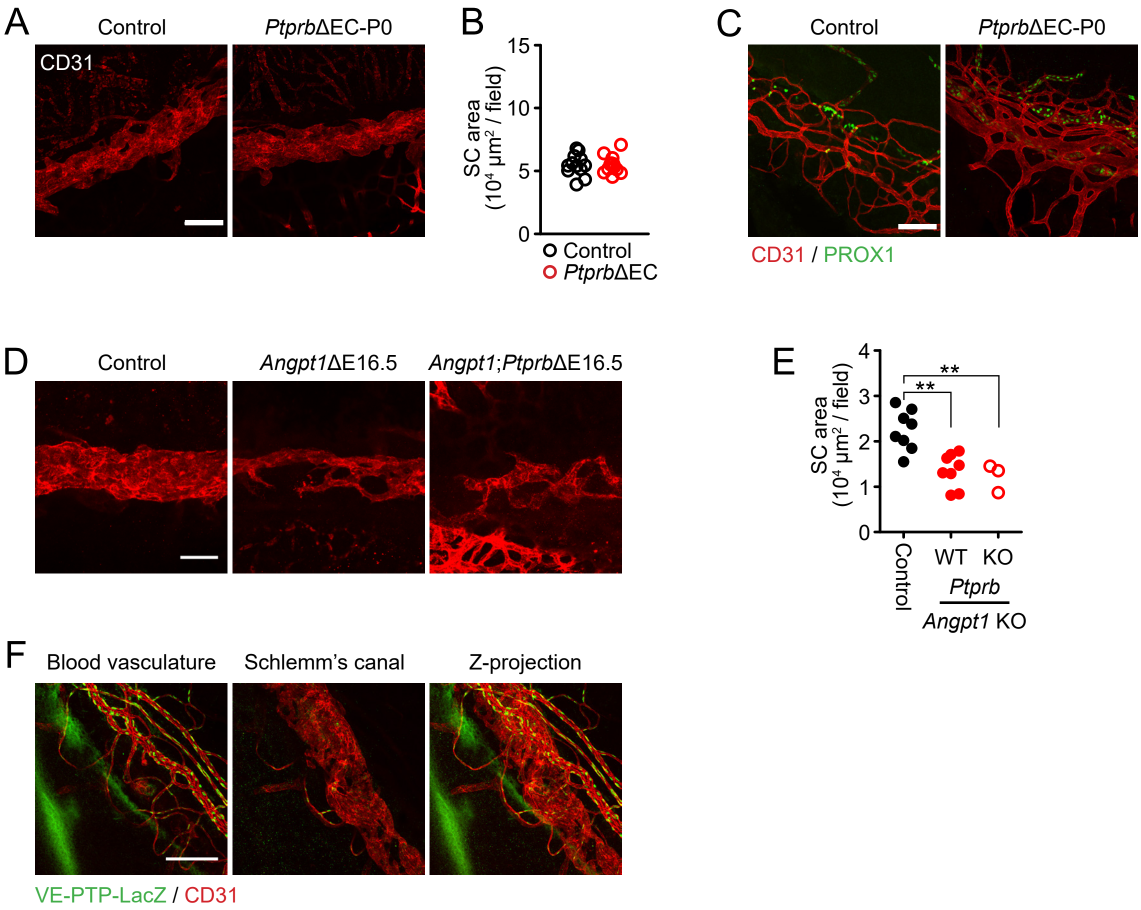

Supplemental Figure 12. (**A**, quantified in **B**) No difference in CD31-positive Schlemm's canal area was observed at P14 in endothelial-specific *Ptprb* knockout mice induced at postnatal day 0 (*Ptprb*ΔEC-P0 mice). (**C**) At P14, *Ptprb*ΔEC-P0 mice exhibited marked dilation of the superficial limbal vasculature although no change in lymphatic morphology was observed. (**D**, quantified in **E**) *Angpt1* whole-body knockout mice induced with doxycycline at embryonic day 16.5 showed a significant reduction in SC area when imaged at P14. However, parallel deletion of Ptprb had no effect on SC area. n = 8 (control), 8 (*Angpt1* KO) and 3 (*Angpt1*;*Pt-prb* double KO). (**F**) Confocal imaging of eyes from *Ptprb*-NLS-LacZ transgenic mice revealed higher β-Gal expression in the limbal blood vessels than in the underlying Schlemm's canal. Scale bars indicate 100 µm (A,B, F) and 50 µm (C). Field size used for SC quantification represents an area of 262,144 µm^2 (B) or 65,096 µm^2 (E). ** $p \leq 0.01$ as determined by 1-way ANOVA with Bonferroni's correction.

Supplemental table 1. Genes linked to glaucoma, ocular hypertension or Schlemm's canal development/function by GWAS, genetic studies or animal models.

Gene	Evidence	Ref
ABCA1	GWAS	Gharahkhani et al. 2014 (1), Bailey et al. 2016 (2), Chen et al. 2014 (3)
ADAMTS2	GWAS	Iglesias et al 2018 (4)
ADAMTS8	GWAS	Iglesias et al 2018 (4)
AFAP1	GWAS	Gharahkhani et al. 2014 (1), MacGregor et al 2018
ANGPT1	GWAS, genetic, animal model	MacGregor et al 2018 (5), Thomson et al. 2017 (6)
ANKH	GWAS	MacGregor et al 2018 (5)
ANKH	GWAS	Khawaja et al. 2018 (7)
ANKH	GWAS	Choquet et al 2018 (8)
ARHGEF12	GWAS	MacGregor et al 2018 (5)
ARVCF	GWAS	Iglesias et al 2018 (4)
ATXN2	GWAS	Bailey et al. 2016 (2)
BCAS3	GWAS	Springelkamp et al. 2017 (9)
BICC1	GWAS	MacGregor et al 2018 (5)
BMP4	Animal models	Chang et al. 2001 (10), van der Merwe, 2016 (11)
CADM2	GWAS	MacGregor et al 2018 (5)
CADM2	GWAS	Choquet et al 2018 (8)
CAV1	GWAS	Thorleifsson, et al. 2010 (12), Hysi et al. 2014 (13)
CAV2	GWAS	MacGregor et al 2018, Thorleifsson, et al. 2010 (12)
CDH11	GWAS	Khawaja et al. 2018(7)
CHAT	GWAS	Khor et al. 2016 (14)
CFTR	GWAS	MacGregor et al 2018 (5)
COL11A1	GWAS	Khor et al. 2016 (14)
COL12A1	GWAS	Iglesias et al 2018 (4)
COL21A1	GWAS	Bonnemaijer et al., 2018 (15)
COL6A2	GWAS	Iglesias et al 2018 (4)
CTTNBP2	GWAS	Khawaja et al. 2018(7), MacGregor et al 2018 (5)
CYP1B1	genetic	Stoilov et al. 1997 (16)
DCN	GWAS	Iglesias et al 2018 (4)
DGKG	GWAS	MacGregor et al 2018 (5)
DGKG	GWAS	Khawaja et al. 2018(7)
DGKG	GWAS	Choquet et al 2018 (8)
EFEMP1	GWAS	Springelkamp et al. 2017 (9)
ENO4	GWAS	Taylor et al 2019 (17)
EPDR1	GWAS	Khor et al. 2016 (14)
ETS1	GWAS	Khawaja et al. 2018(7), MacGregor et al 2018 (5)
EXOC2	GWAS	Khawaja et al. 2018(7), MacGregor et al 2018 (5), Choquet et al 2018 (8)
EXOC4	GWAS	Bonnemaijer et al., 2018 (15)
FANCA	GWAS	Khawaja et al. 2018(7)
FBN1	GWAS	Iglesias et al 2018 (4)
FBXO32	GWAS	Khawaja et al. 2018(7)
FERMT2	GWAS	Khor et al. 2016 (14)
FGF1	GWAS	Iglesias et al 2018 (4)
FLT1	Animal model	Sano et al. 2012 (18)
FLT4	Animal model	Aspelund et al. 2014 (19)
FMNL2	GWAS	Choquet et al 2018 (8)
FNDC3B	GWAS	Hysi et al. 2014 (13)

Supplemental table 1. Genes linked to glaucoma, ocular hypertension or Schlemm's canal development/function by GWAS, genetic studies or animal models. (continued)

Gene	Evidence	Ref
FNDC3E	GWAS	Shiga et al. 2018 (20)
FOXC1	GWAS	Bailey et al. 2016 (2)
GAS7	GWAS	Hysi et al. 2014 (13), MacGregor et al 2018
GLIS3	GWAS	Iglesias et al 2018 (4), Khor et al. 2016 (14)
GMDS	GWAS	Gharahkhani et al. 2014 (1), MacGregor et al 2018
HABP2	GWAS	Iglesias et al 2018 (4)
HMGA2	GWAS	Shiga et al. 2018 (20)
IKZF2	GWAS	Choquet et al 2018 (8)
KBTBD8	GWAS	Khawaja et al. 2018(7)
KDR	Animal model	Sano et al. 2012 (18), Kizhatil et al. 2014 (21)
LHPF	GWAS	Shiga et al. 2018 (20)
LMX1B	GWAS	MacGregor et al 2018 (5)
LMX1B	GWAS	Khawaja et al. 2018(7)
LMX1B	GWAS	Choquet et al 2018 (8)
LMX1B	GWAS	Shiga et al. 2018 (20)
LOXL1	GWAS	Shiga et al. 2018 (20)
LOXL2	GWAS	Iglesias et al 2018 (4)
LRIG1	GWAS	Khawaja et al. 2018(7)
LTBP1	GWAS	Iglesias et al 2018 (4)
LTBP2	Genetic	Ali et al. 2009 (22)
ME3	GWAS	Khawaja et al. 2018(7)
MECOM	GWAS	MacGregor et al 2018 (5)
MEIS2	GWAS	Shiga et al. 2018 (20)
MYOC	Genetic	Kaur et al. 2005 (23)
MYOF	GWAS	MacGregor et al 2018 (5)
NDUFAF6	GWAS	Iglesias et al 2018 (4)
PDE7B	GWAS	Khawaja et al. 2018(7)
PDE7B	GWAS	Choquet et al 2018 (8)
PKHD1	GWAS	Khawaja et al. 2018(7)
PLEKHA7	GWAS	Khor et al. 2016 (14)
PMM2	GWAS	Chen et al. 2014 (3)
PTPRJ	GWAS	Hysi et al. 2014 (13)
RARB	GWAS	Springelkamp et al. 2017 (9)
RAPSN	GWAS	Hysi et al. 2014 (13)
RSPO1	GWAS	Khawaja et al. 2018(7)
RUNX2	GWAS	Iglesias et al 2018 (4)
SAMD9	GWAS	Iglesias et al 2018 (4)
SIX6	GWAS	MacGregor et al 2018 (5)
STAG1	GWAS	Iglesias et al 2018 (4)
STON2	GWAS	Iglesias et al 2018 (4)
SVEP1	Genetic, GWAS	Young et al. 2020 (24), Gharahkhani et al. 2020 (25)
TBCCD1	GWAS	MacGregor et al 2018 (5)
TEK	Genetic, animal model	Souma et al. 2016 (26)
TGFB2	GWAS	Iglesias et al 2018 (4)
TGFBR3	GWAS	Springelkamp et al. 2017 (9)
THBS2	GWAS	Iglesias et al 2018 (4)

Supplemental table 1. Genes linked to glaucoma, ocular hypertension or Schlemm's canal development/function by GWAS, genetic studies or animal models. (continued)

Gene	Evidence	Ref
THSD7A	GWAS	MacGregor et al 2018 (5)
TMCO1	GWAS	Burdon et al. 2011 (27), Hysi et al. 2014 (13)
TMCT2	GWAS	Choquet et al 2018 (8)
TXNRD2	GWAS	Bailey et al. 2016 (2), MacGregor et al 2018 (5)
VEGFA	Animal model	Aspelund et al. 2014 (19)
VEGFC	Animal model	Aspelund et al. 2014 (19)
VPS13C	GWAS	Khawaja et al. 2018(7)

References for Supplemental table 1

1. Gharahkhani P, Burdon KP, Fogarty R, Sharma S, Hewitt AW, Martin S, et al. Common variants near ABCA1, AFAP1 and GMDS confer risk of primary open-angle glaucoma. Nat Genet. 2014;46(10):1120-5.

2. Bailey JNC, Loomis SJ, Kang JH, Allingham RR, Gharahkhani P, Khor CC, et al. Genome-wide association analysis identifies TXNRD2, ATXN2 and FOXC1 as susceptibility loci for primary open-angle glaucoma. Nat Genet. 2016;48(2):189-94.

3. Chen Y, Lin Y, Vithana EN, Jia L, Zuo X, Wong TY, et al. Common variants near ABCA1 and in PMM2 are associated with primary open-angle glaucoma. Nat Genet. 2014;46(10):1115-9.

4. Iglesias AI, Mishra A, Vitart V, Bykhovskaya Y, Höhn R, Springelkamp H, et al. Cross-ancestry genome-wide association analysis of corneal thickness strengthens link between complex and Mendelian eye diseases. Nature Communications. 2018;9(1):1864.

5. MacGregor S, Ong J-S, An J, Han X, Zhou T, Siggs OM, et al. Genome-wide association study of intraocular pressure uncovers new pathways to glaucoma. Nat Genet. 2018;50(8):1067-71.

6. Thomson BR, Souma T, Tompson SW, Onay T, Kizhatil K, Siggs OM, et al. Angiopoietin-1 is required for Schlemm's canal development in mice and humans. J Clin Invest. 2017;127(12):4421-36.

7. Khawaja AP, Cooke Bailey JN, Wareham NJ, Scott RA, Simcoe M, Igo RP, et al. Genome-wide analyses identify 68 new loci associated with intraocular pressure and improve risk prediction for primary open-angle glaucoma. Nat Genet. 2018;50(6):778-82.

8. Choquet H, Paylakhi S, Kneeland SC, Thai KK, Hoffmann TJ, Yin J, et al. A multiethnic genome-wide association study of primary open-angle glaucoma identifies novel risk loci. Nature Communications. 2018;9(1):2278.

9. Springelkamp H, Iglesias AI, Mishra A, Höhn R, Wojciechowski R, Khawaja AP, et al. New insights into the genetics of primary open-angle glaucoma based on meta-analyses of intraocular pressure and optic disc characteristics. Hum Mol Genet. 2017;26(2):438-53.

10. Chang B, Smith RS, Peters M, Savinova OV, Hawes NL, Zabaleta A, et al. Haploinsufficient Bmp4 ocular phenotypes include anterior segment dysgenesis with elevated intraocular pressure. BMC Genet. 2001;2(1):18.

11. van der Merwe EL, and Kidson SH. Wholemount imaging reveals abnormalities of the aqueous outflow pathway and corneal vascularity in Foxc1 and Bmp4 heterozygous mice. Exp Eye Res. 2016;146:293-303.

12. Thorleifsson G, Walters GB, Hewitt AW, Masson G, Helgason A, DeWan A, et al. Common variants near CAV1 and CAV2 are associated with primary open-angle glaucoma. Nat Genet. 2010;42(10):906-9.

References for Supplemental table 1, continued

13. Hysi PG, Cheng C-Y, Springelkamp H, Macgregor S, Bailey JNC, Wojciechowski R, et al. Genome-wide analysis of multi-ancestry cohorts identifies new loci influencing intraocular pressure and susceptibility to glaucoma. Nat Genet. 2014;46(10):1126-30.

14. Khor CC, Do T, Jia H, Nakano M, George R, Abu-Amero K, et al. Genome-wide association study identifies five new susceptibility loci for primary angle closure glaucoma. Nat Genet. 2016;48(5):556-62.

15. Bonnemaijer PWM, Iglesias AI, Nadkarni GN, Sanyiwa AJ, Hassan HG, Cook C, et al. Genome-wide association study of primary open-angle glaucoma in continental and admixed African populations. Hum Genet. 2018;137(10):847-62.

16. Stoilov I, Akarsu AN, and Sarfarazi M. Identification of Three Different Truncating Mutations in Cytochrome P4501B1 (CYP1B1) as the Principal Cause of Primary Congenital Glaucoma (Buphthalmos) in Families Linked to the GLC3A Locus on Chromosome 2p21. Hum Mol Genet. 1997;6(4):641-7.

17. Taylor KD, Guo X, Zangwill LM, Liebmann JM, Girkin CA, Feldman RM, et al. Genetic Architecture of Primary Open-Angle Glaucoma in Individuals of African Descent: The African Descent and Glaucoma Evaluation Study III. Ophthalmology. 2019;126(1):38-48.

18. Sano K, Katsuta O, Shirae S, Kubota Y, Ema M, Suda T, et al. Flt1 and Flk1 mediate regulation of intraocular pressure and their double heterozygosity causes the buphthalmia in mice. Biochem Biophys Res Commun. 2012;420(2):422-7.

19. Aspelund A, Tammela T, Antila S, Nurmi H, Lepp, xE, et al. The Schlemm's canal is a VEGF-C/VEGFR-3–responsive lymphatic-like vessel. J Clin Invest. 2014;124(9):3975-86.

20. Shiga Y, Akiyama M, Nishiguchi KM, Sato K, Shimozawa N, Takahashi A, et al. Genome-wide association study identifies seven novel susceptibility loci for primary open-angle glaucoma. Hum Mol Genet. 2018;27(8):1486-96.

21. Kizhatil K, Ryan M, Marchant JK, Henrich S, and John SWM. Schlemm's Canal Is a Unique Vessel with a Combination of Blood Vascular and Lymphatic Phenotypes that Forms by a Novel Developmental Process. PLoS Biol. 2014;12(7):e1001912.

22. Ali M, McKibbin M, Booth A, Parry DA, Jain P, Riazuddin SA, et al. Null Mutations in LTBP2 Cause Primary Congenital Glaucoma. The American Journal of Human Genetics. 2009;84(5):664-71.

23. Kaur K, Reddy A, Mukhopadhyay A, Mandal A, Hasnain S, Ray K, et al. Myocilin gene implicated in primary congenital glaucoma. 2005;67(4):335-40.

24. Young TL, Whisenhunt KN, Jin J, LaMartina SM, Martin SM, Souma T, et al. SVEP1 as a Genetic Modifier of TEK-Related Primary Congenital Glaucoma. Invest Ophthalmol Vis Sci. 2020;61(12):6-.

25. Gharahkhani P, Jorgenson E, Hysi P, Khawaja AP, Pendergrass S, Han X, et al. A large cross-ancestry meta-analysis of genome-wide association studies identifies 69 novel risk loci for primary open-angle glaucoma and includes a genetic link with Alzheimer's disease. bioRxiv. 2020:2020.01.30.927822.

26. Souma T, Tompson SW, Thomson BR, Siggs OM, Kizhatil K, Yamaguchi S, et al. Angiopoietin receptor TEK mutations underlie primary congenital glaucoma with variable expressivity. J Clin Invest. 2016;126(7):2575-87.

27. Burdon KP, Macgregor S, Hewitt AW, Sharma S, Chidlow G, Mills RA, et al. Genome-wide association study identifies susceptibility loci for open angle glaucoma at TMCO1 and CDKN2B-AS1. Nat Genet. 2011;43(6):574-8.

Supplemental table 2. PCR primer sequences used in the study

Primer name	Sequence	Product Length
Mouse qRT-PCR *Angpt1*-Fwd	5'-GGGGGAGGTTGGACAGTAA	174bp
Mouse qRT-PCR *Angpt1*-Rev	5'-CATCAGCTCAATCCTCAGC	
Mouse qRT-PCR *Gapdh*-Fwd	5'- AAGGTCATCCCAGAGCTGAA	138bp
Mouse qRT-PCR *Gapdh*-Rev	5'-CTGCTTCACCACCTTCTTGA	
Genotyping *Angpt1*-flox Fwd	5'-CAATGCCAGAGGTTCTTGTGAA	Floxed: 328bp / WT: 233
Genotyping *Angpt1*-flox Rev	5'-TCAAAGCAACATATCATGTGCA	
Genotyping *Wnt1*-Cre Fwd	5'- CAGCGCCGCAACTATAAGAG	305bp
Genotyping *Wnt1*-Cre Rev	5'- CATCGACCGGTAATGCAG	
Genotyping Cre Fwd	5'- GTGCAAGTTGAATAACCGGAAATGG	673bp
Genotyping Cre Rev	5'-AGAGTCATCCTTAGCGCCGTAAATCAAT	
Genotyping *Svep1*-Flox Fwd	5'- GGTAGTTTCTGCCCCAGTCAGC	Floxed: 328bp / WT: 233
Genotyping *Svep1*-Flox Rev	5'- CTTTTCTTTGCCTGCCTTAATGCC	
Genotyping *Svep1*-Del Fwd	5'-GGTAGTTTCTGCCCCAGTCAGC	Del: 456bp / WT: 1128
Genotyping *Svep1*-Del Rev	5'- TTCAGATTTGGTCAAAATGCAGTCC	
Genotyping *Ptprb*-Flox Fwd	5'-GCGTCTATCCAGTGGAGGACTTTC	Floxed: 328bp / WT: 233
Genotyping *Ptprb*-Flox Rev	5'-CCAGGTGCCGTTCATTCAGC	
Genotyping *Rosa26*-rtTA A	5'- AAGGGAGCTGCAGTGGAGTA	rtTA: 650bp / WT: 500
Genotyping *Rosa26*-rtTA B	5'- GGCGAGTTTACGGGTTGTTA	
Genotyping *Rosa26*-rtTA C	5'- TCCGAGGCGGATCACAAGCA	
