# Supplementary material for: Cellular crosstalk regulates the aqueous humor outflow pathway and provides new targets for glaucoma therapies
Source: Nat Commun. 2021 Oct 18;12:6072. doi: 10.1038/s41467-021-26346-0 (PMC8523664; doi:10.1038/s41467-021-26346-0)
Supplement: Supplementary file 4 — Description of Additional Supplementary Files [file 41467_2021_26346_MOESM4_ESM.pdf]

Title: Supplemental dataset 1.

Description: Differential expression analysis after single cell RNA sequencing of 6 week old wild-type mouse limbus samples allowed identification of specific cell clusters. Differential expression was calculated for each cluster compared to the full wild-type dataset (as described in Figure 5), within the endothelial cell population (Figure 6) and within the trabecular meshwork-containing clusters (Figure 7) using MAST. For all clusters, the top 100 differentially expressed genes are included, ranked by Log_2-fold change. Cluster: Cell cluster under comparison, as described in figures 5-7. avg_log2FC_vs_full_dataset: Log_2-fold change of average within-cluster gene expression as compared to the whole dataset, all endothelial cells or all trabecular-meshwork-containing clusters. p_val/p_val_corrected: Calculated p value/Bonferroni corrected p value of the comparison. pct_of_cells_in_cluster_expressing_gene: Proportion of droplets within the cluster where the transcript is detected. pct_of_cells_in_dataset_expressing_gene: Proportion of droplets within the full dataset where the transcript is detected.

Title: Supplemental dataset 2.

Description: Averaged normalized expression (arbitrary units) of all transcripts detected in 6 week old, wild-type mouse Schlemm's canal endothelial cells or trabecular meshwork cells (clusters TM_3, TM_4, TM_5, TM_7 and TM_11). Transcripts with normalized expression >0.3 arb. units are shown and were used for identification of putative TM-SC crosstalk pathways.
