# Supplementary material for: Cellular crosstalk regulates the aqueous humor outflow pathway and provides new targets for glaucoma therapies
Source: Nat Commun. 2021 Oct 18;12:6072. doi: 10.1038/s41467-021-26346-0 (PMC8523664; doi:10.1038/s41467-021-26346-0)
Supplement: Supplementary file 5 — Reporting Summary [file 41467_2021_26346_MOESM5_ESM.pdf]

nature research

Corresponding author(s): Susan E Quaggin
Last updated by author(s): Aug 9, 2021

Reporting Summary

Nature Research wishes to improve the reproducibility of the work that we publish. This form provides structure for consistency and transparency in reporting. For further information on Nature Research policies, see our Editorial Policies and the Editorial Policy Checklist.

Statistics

For all statistical analyses, confirm that the following items are present in the figure legend, table legend, main text, or Methods section.

n/a	Confirmed	
☐	☒	The exact sample size (n) for each experimental group/condition, given as a discrete number and unit of measurement
☐	☒	A statement on whether measurements were taken from distinct samples or whether the same sample was measured repeatedly
☐	☒	The statistical test(s) used AND whether they are one- or two-sided<br>*Only common tests should be described solely by name; describe more complex techniques in the Methods section.*
☐	☒	A description of all covariates tested
☐	☒	A description of any assumptions or corrections, such as tests of normality and adjustment for multiple comparisons
☐	☒	A full description of the statistical parameters including central tendency (e.g. means) or other basic estimates (e.g. regression coefficient) AND variation (e.g. standard deviation) or associated estimates of uncertainty (e.g. confidence intervals)
☐	☒	For null hypothesis testing, the test statistic (e.g. F, t, r) with confidence intervals, effect sizes, degrees of freedom and P value noted<br>*Give P values as exact values whenever suitable.*
☒	☐	For Bayesian analysis, information on the choice of priors and Markov chain Monte Carlo settings
☒	☐	For hierarchical and complex designs, identification of the appropriate level for tests and full reporting of outcomes
☒	☐	Estimates of effect sizes (e.g. Cohen's d, Pearson's r), indicating how they were calculated

Our web collection on statistics for biologists contains articles on many of the points above.

Software and code

Policy information about availability of computer code

Data collection	Image quantification was performed using ImageJ Fiji 2.0 software running on ImageJ 1.53c and single cell RNA sequencing was performed using the 10x Genomics platform.
Data analysis	RNA sequencing data was analyzed using 10x Genomics Cellranger 3, R version 3.6.1, Seurat 3.1.3, scDblFinder 1.4.0 and maxLik 1.4.8.<br>Custom R code is available via GitHub at https://github.com/benrthomson/Angpt1.Angle.SingleCell (https://doi.org/10.5281/zenodo.5172757)<br>Statistical analysis was conducted using Graphpad Prism 5.

For manuscripts utilizing custom algorithms or software that are central to the research but not yet described in published literature, software must be made available to editors and reviewers. We strongly encourage code deposition in a community repository (e.g. GitHub). See the Nature Research guidelines for submitting code & software for further information.

Data

Policy information about availability of data

All manuscripts must include a data availability statement. This statement should provide the following information, where applicable:

- Accession codes, unique identifiers, or web links for publicly available datasets
- A list of figures that have associated raw data
- A description of any restrictions on data availability

Single cell sequencing data associated with Figures 5-7, Table 1 and Supplemental Datasets 1 and 2 is available on the NCBI Gene Expression Omnibus (GEO, accession number GSE168200 [https://www.ncbi.nlm.nih.gov/geo/query/acc.cgi?acc=GSE168200]). Source data for all figures are provided with this paper.

Field-specific reporting

Please select the one below that is the best fit for your research. If you are not sure, read the appropriate sections before making your selection.

☒ Life sciences ☐ Behavioural & social sciences ☐ Ecological, evolutionary & environmental sciences

For a reference copy of the document with all sections, see nature.com/documents/nr-reporting-summary-flat.pdf

Life sciences study design

All studies must disclose on these points even when the disclosure is negative.

Sample size	For studies of Angpt1 and Svep1 knockout mice, power analysis was performed to estimate animal numbers required to detect biologically relevant changes in intraocular pressure and Schlemm's canal area. Litters of animals were then generated, using those estimates to guide breeding. Generated animals were then included in the study on a whole-litter basis, so that no mutants were included without matching littermate controls. For studies of IOP in C57bl/6J wildtype mice, power analysis was used to estimate animal numbers required to obtain a biologically meaningful reduction in IOP.
Data exclusions	During analysis of single cell RNA sequencing data, low-quality cells were filtered from the dataset using commonly-accepted methods based on mitochondrial RNA percentage and predicted doublet status as described in the manuscript and supplemental figure 5. In figure 8i, a single retina was excluded from analysis due to poor staining. No data was excluded from other results.
Replication	Animal experiments were conducted on groups consisting of multiple independent litters measured in parallel as described in the manuscript. All data included in the manuscript was successfully replicated in multiple animal groups with the exception of scRNAseq data which was limited to a single experiment for reasons of cost, and hepta-ANGPT1 treatment of wildtype C57Bl/6J mice which was conducted on a single cohort of adult animals.
Randomization	For genetic studies of knockout vs control animals, randomization was not possible as group assignment was determined by genotype. For drug treatment studies, litters were randomized into treatment and control groups at birth prior to initiating the experiment.
Blinding	IOP measurement, tissue collection, real-time PCR, histology, imaging and image quantification were performed in a blinded fashion. Blinding was not possible during scRNAseq analysis as lack of Angpt1 expression quickly identified the knockout dataset. Other experiments were not blinded.

Reporting for specific materials, systems and methods

We require information from authors about some types of materials, experimental systems and methods used in many studies. Here, indicate whether each material, system or method listed is relevant to your study. If you are not sure if a list item applies to your research, read the appropriate section before selecting a response.

Materials & experimental systems

n/a	Involved in the study	
☐	☒	Antibodies
☒	☐	Eukaryotic cell lines
☒	☐	Palaeontology and archaeology
☐	☒	Animals and other organisms
☒	☐	Human research participants
☒	☐	Clinical data
☒	☐	Dual use research of concern

Methods

n/a	Involved in the study	
☒	☐	ChIP-seq
☒	☐	Flow cytometry
☒	☐	MRI-based neuroimaging

Antibodies

Antibodies used	Mouse anti-BRN3A (Millipore; MAB1585; Lot 2683938, Clone # 5A3.2)<br>Goat anti-BRN3B (Santa Cruz Biotechnology; sc-6026; Lot K0215, polyclonal)<br>Rabbit anti-TUBB3 (Covance; MRB-435P; Lot unknown; Clone # TUJ1 1-15-79)<br>Rat anti mouse CD31 antibody (BD; #553370; Lot #8043575; Clone MEC13.3)<br>Goat anti-PROX1 antibodies (R&D systems AF2727; Lot #V1Y0216011; Polyclonal)<br>Goat anti-VEGFR3 (R&D systems #AF349; Lot #DAB0214081; Polyclonal)<br>Goat anti-mouse CD31 (R&D systems; #AF3628; Lot #YZU0118051; Polyclonal)<br>Rabbit anti-human SVEP1 (Aviva; #ARP58239; Lot #QC24168; Polyclonal)<br>Alexafluor-594 conjugated Donkey anti Rat igG (ThermoFisher #A21209; Lot #2078918; Polyclonal)<br>Alexafluor-488 conjugated Donkey anti goat igG (ThermoFisher #A11055; Lot #1869589; Polyclonal)<br>Alexafluor-488 conjugated Donkey anti mouse igG (ThermoFisher #A21202 Lot #1741782; Polyclonal)<br>Alexafluor-647 conjugated Donkey anti goat igG (ThermoFisher #A21447 Lot #2273668; Polyclonal)

Validation	Mouse anti-BRN3A (Millipore; MAB1585; Lot 2683938, Clone # 5A3.2)<br>Manufacturer reports binding to chicken, monkey and rat, widely published in mouse.<br>E.g: https://doi.org/10.1002/cne.23521<br><br>Goat anti-BRN3B (Santa Cruz Biotechnology; sc-6026; Lot K0215, polyclonal)<br>Manufacturer reports binding to human Brn3b, widely published in mouse.<br>E.g: https://doi.org/10.1002/cne.22765<br><br>Rabbit anti-TUBB3 (Covance; MRB-435P; Lot unknown; Clone # TUJ1 1-15-79)<br>Reported reactivity to: Human, Mouse, Rat, Bovine, Canine, Donkey, Feline, Goat, Guinea Pig, Hamster, Horse, Porcine, Rabbit, Sheep, Simian<br><br>Rat anti mouse CD31 antibody (BD; #553370; Lot #8043575; Clone MEC13.3)<br>Widely published, manufacturer reported reactivity to mouse CD31/Pecam1.<br><br>Goat anti-PROX1 antibodies (R&D systems AF2727; Lot #V1Y0216011; Polyclonal)<br>Manufacturer reports reactivity against human PROX1, widely reported in mouse, including https://doi.org/10.1038/s41586-020-2998-x<br><br>Goat anti-VEGFR3 (R&D systems #AF349; Lot #DAB0214081; Polyclonal)<br>Manufacturer reports reactivity against human VEGFR3, widely reported in mouse, including https://doi.org/10.1038/s41586-020-2998-x<br><br>Goat anti-mouse CD31 (R&D systems; #AF3628; Lot #YZU0118051; Polyclonal)<br>Manufacturer reports reactivity against Mouse/Rat CD31, validated against CD31 of human origin by the Mouse phenotyping and histology core of the Feinberg School of Medicine.<br><br>Rabbit anti-human SVEP1 (Aviva; #ARP58239; Lot #QC24168; Polyclonal)<br>Manufacturer reports reactivity against human SVEP1

Animals and other organisms

Policy information about studies involving animals; ARRIVE guidelines recommended for reporting animal research

Laboratory animals	Mice used in the study were of mixed sex on a mixed genetic background as detailed in the manuscript with the exception of male animals used for adult hepta ANGPT1 treatment (figure 8 J). These animals were on a pure C57Bl6/J background and were purchased from the Jackson Laboratory as described in the manuscript. Ages of all experimental animals is reported in the manuscript.
Wild animals	No wild animals were used
Field-collected samples	No field collected samples were used.
Ethics oversight	Animal experiments were approved by the Animal Care and Use Committee at Northwestern University.

Note that full information on the approval of the study protocol must also be provided in the manuscript.
